# Tracking heterogeneous structural motifs and the redox behaviour of copper–zinc nanocatalysts for the electrocatalytic CO_2_ reduction using operando time resolved spectroscopy and machine learning†

**DOI:** 10.1039/d2cy00227b

**Published:** 2022-03-10

**Authors:** Martina Rüscher, Antonia Herzog, Janis Timoshenko, Hyo Sang Jeon, Wiebke Frandsen, Stefanie Kühl, Beatriz Roldan Cuenya

**Affiliations:** Department of Interface Science, Fritz-Haber Institute of the Max-Planck Society 14195 Berlin Germany janis@fhi-berlin.mpg.de roldan@fhi-berlin.mpg.de

## Abstract

Copper-based catalysts are established catalytic systems for the electrocatalytic CO_2_ reduction reaction (CO_2_RR), where the greenhouse gas CO_2_ is converted into valuable industrial chemicals, such as energy-dense C_2+_ products, using energy from renewable sources. However, better control over the catalyst selectivity, especially at industrially relevant high current density conditions, is needed to expedite the economic viability of the CO_2_RR. For this purpose, bimetallic materials, where copper is combined with a secondary metal, comprise a promising and a highly tunable catalyst for the CO_2_RR. Nevertheless, the synergy between copper and the selected secondary metal species, the evolution of the bimetallic structural motifs under working conditions and the effect of the secondary metal on the kinetics of the Cu redox behavior require careful investigation. Here, we employ *operando* quick X-ray absorption fine structure (QXAFS) spectroscopy coupled with machine-learning based data analysis and surface-enhanced Raman spectroscopy (SERS) to investigate the time-dependent chemical and structural changes in catalysts derived from shape-selected ZnO/Cu_2_O nanocubes under CO_2_RR conditions at current densities up to −500 mA cm^−2^. We furthermore relate the catalyst transformations observed under working conditions to the catalytic activity and selectivity and correlate potential-dependent surface and subsurface processes. We report that the addition of Zn to a Cu-based catalyst has a crucial impact on the kinetics of subsurface processes, while redox processes of the Cu surface layer remain largely unaffected. Interestingly, the presence of Zn was found to contribute to the stabilization of cationic Cu(i) species, which is of catalytic relevance since Cu(0)/Cu(i) interfaces have been reported to be beneficial for efficient electrocatalytic CO_2_ conversion to complex multicarbon products. At the same time, we attribute the increase of the C_2+_ product selectivity to the formation of Cu-rich CuZn alloys in samples with low Zn content, while Zn-rich alloy phases result in an increased formation of CO paralleled by an increase of the parasitic hydrogen evolution reaction.

## Abstract 2

Elucidating the role of Zn in bimetallic CuZn nanocatalysts for the electrocatalytic CO_2_ reduction reaction (CO_2_RR), where the greenhouse gas CO_2_ is converted into valuable industrial chemicals using energy from renewable sources.

## Graphical abstract

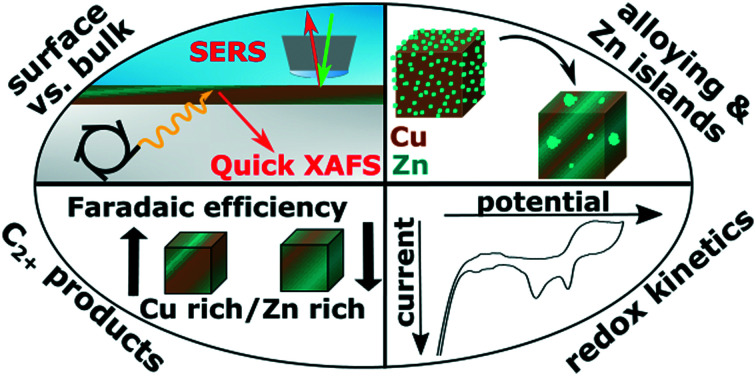

## 1 Introduction

The electrochemical CO_2_ reduction reaction (CO_2_RR) is among the most promising electrocatalytic reactions for emerging energy schemes. Within the framework of the carbon-neutrality strategy, the CO_2_RR shows promise for transforming the greenhouse gas CO_2_ into valuable chemical feedstocks and fuels using electricity from renewable energy sources, thus decreasing our dependency on fossil fuel resources. Nevertheless, for practical applications of the CO_2_RR, a better control of the product selectivity,^1^ the reaction kinetics^2^ and the catalyst stability^3,4^ needs to be achieved. So far, the majority of the current studies on the CO_2_RR have been carried out in aqueous-fed electrochemical cells (such as H-type cells), where the current density is limited to a few mA cm^−2^ due to the low CO_2_ solubility and mass-transport limitations.^5^ However, commercially viable applications require the optimization of the CO_2_RR with current densities well above −200 mA cm^−2^.^6,7^ Such current densities can be reached in gas-fed flow cells, where CO_2_ is supplied through a gas diffusion electrode (GDE). The effect of the harsh experimental conditions associated with the high current densities on the catalyst structure still remains poorly understood, since only few *operando* investigations have been carried out under these conditions.^8^ Moreover, even at lower current densities the interpretation of the catalyst structure and its relation to the catalytic functionality remains a challenge due to the coexistence of different species in the catalyst and their potential-dependent evolution under working conditions. This is especially true for more complex catalytic systems containing metals of different types, or those for which numerous combinations of various crystallographic structures, metal oxidation states and degrees of alloying might coexist under reaction conditions. The contributions of these different species are often very challenging to disentangle, especially if the low spectroscopic contrast between those species hinders their reliable identification in experimental data.

In the context of the CO_2_RR, Cu-based catalysts have played a prominent role, as Cu is the only known transition metal capable of converting CO_2_ to energy-dense C_2+_ products with sufficient yield.^9^ A common route to further tune the product selectivity of Cu-based catalysts is the combination of copper with a secondary metal. Here, a popular option is to use a metal that can efficiently convert CO_2_ into CO (*e.g.*, Ag,^10^ Au (ref. 11 and 12) or Zn (ref. 13–16)). Zn is of special interest for the development of practical bimetallic CO_2_RR catalysts, since it is abundant and inexpensive compared to Ag and Au.^16^ On one hand, the CO produced at the Zn sites might get dimerized at the Cu sites (the so called “spillover effect”), boosting the production of C_2+_ products.^15^ On the other hand, the presence of bimetallic structural motifs, and changes in the Cu lattice spacing due to alloying can affect the adsorption energies of reaction intermediates.^17^ These effects, for instance, can suppress the parasitic hydrogen evolution reaction (HER), thus resulting in a more efficient CO_2_RR process.^13,18,19^ While several studies on CuZn electrocatalysts report high stability of the CO_2_RR catalytic performance over several hours,^15,20–22^ it has also been shown that significant changes in the CuZn catalyst performance can occur over time. The catalyst evolution, however, strongly depends on its composition,^19^ particle size and shape.^14,20^ The changes can be associated with morphological, structural and chemical transformations. Our incomplete understanding of these processes hinders the design of novel CO_2_RR catalysts. There, the restructuring of the catalyst due to alloying processes,^23^ different reduction rates for CuO_*x*_ and ZnO species and their different stability contribute to the time dependencies of the activity and selectivity under reaction conditions.^13^ Therefore, it is imperative to monitor the presence of the desired bimetallic motifs under catalysts' working conditions by using suitable *operando* techniques, such as X-ray absorption spectroscopy (XAS).^24,25^ In particular, the high sensitivity of the *operando* XAS method toward the local structure and the chemical state as well as its element-specificity provide unique advantages for understanding the structure and composition of multi-metal catalysts.^26,27^ Moreover, the application of the quick X-ray absorption fine structure (QXAFS) method, which allows the collection of XAS data with subsecond time-resolution, has recently gained significance as an invaluable tool for understanding the catalyst evolution with time, both under static and dynamic reaction conditions.^8,28^ In particular, in depth analysis of time-resolved X-ray absorption near-edge structure (XANES) and extended X-ray absorption fine structure (EXAFS) spectra^26^ is key for the understanding of the dynamic changes that electrocatalytic materials undergo under working conditions.

In this study, we applied *operando* QXAFS to elucidate the different roles played by the secondary element (Zn) in our bimetallic CuZn nanocatalysts for the CO_2_RR. Our catalysts are derived from Cu_2_O nanocubes (NCs) and decorated with different amounts of Zn. Using QXAFS, we were able to track their evolution at high current densities and under dynamically changing potentials (cyclic voltammetry (CV) scans). Moreover, we address one of the fundamental limitations of the XAS method, namely its inability to distinguish between different environments around the absorbing atom, if those are comprised of atoms that are neighbours in the periodic table, due to small differences in their photoelectron scattering properties. Here, we show that such a discrimination can, nonetheless, be achieved by careful analysis of XANES data and by applying machine learning approaches for the analysis of EXAFS data.^19,29^ In this case, the analysis must be extended beyond the contribution of the first coordination shell and must exploit the difference in crystallographic structures for Cu-rich phases (fcc-type structure) and for Zn-rich phases (non-fcc type structures).

By using a synergistic combination of *operando* QXAFS, XANES analysis and machine learning, we demonstrate that (i) an increase in the Faradaic efficiency (FE) of the HER with time can be linked to the time dependent evolution of Zn-rich alloy phases with non-fcc structure, which are present especially in bimetallic samples with high Zn loadings, while (ii) Cu-rich CuZn alloys with fcc-type structure favour the formation of C_2+_ products. We also observe that (iii) the presence of Zn species strongly influences the kinetics of the Cu oxidation and reduction. This finding is particularly intriguing, considering that the stabilization of cationic Cu species under CO_2_RR conditions has been suggested as one of the key factors for efficient CO_2_ conversion to complex hydrocarbons.^30,31^ Moreover, by complementing the bulk-sensitive *operando* QXAFS with the *operando* surface-enhanced Raman spectroscopy (SERS) method,^15,32^ we correlate the processes on the catalyst surface and in the sub-surface. Thus, we were able to reveal that (iv) the oxidation and reduction of Cu species proceeds differently for surface oxide species and oxides in the catalyst bulk.

## 2 Methods/experimental

### Sample preparation and *ex situ* characterization

The Zn-decorated Cu_2_O NCs were prepared by a wet-chemical ligand-free method (see ESI†) and were spray-coated on a carbon-based GDE (Sigracet 28BC). The Cu : Zn ratio was later quantified by comparing the absorption edge steps in X-ray absorption data at the Cu K-edge and the Zn K-edge, acquired in transmission mode. For the three bimetallic samples that were investigated, the obtained Zn percentages were 4%, 7% and 15%. The *ex situ* characterization of the NCs was done using a scanning transmission electron microscope (STEM, FEI Talos F200X microscope, Thermo Fisher Scientific) and an energy dispersive spectroscopy (EDX, SuperX 4 SDD EDX detector) featuring an XFEG field emission gun (200 kV) to determine the morphology and elemental distribution of the samples. The detectors in use were bright-field (BF), dark field (DF) and high angle annular dark-field (HAADF) detectors.

### Electrochemical characterization

The electrochemical measurements were carried out in an in-house built gas-fed cell described in our previous study.^8^ To control the potential, a potentiostat (Autolab PGSTAT302N) was used. For all electrochemical measurements, CO_2_-saturated 1 M KHCO_3_ was used as electrolyte. In between catholyte and anolyte, an anion exchange membrane (Selemion AMV, AGC Inc.) was placed. A leak-free Ag/AgCl (Innovative Instruments) was used as reference electrode and a Pt mesh (MaTecK) as counter electrode. In this work, all potentials presented are referenced to the reversible hydrogen electrode (RHE, see Note 3 in ESI†). The exposed area of the working electrode was 1 cm^2^. The selectivity studies were conducted at current densities of either −250 mA cm^−2^ or of −500 mA cm^−2^. For each measurement, a fresh sample was used. The respective current density was held constant for 60 min. Every 15 minutes, the gas products were measured with an online gas chromatograph (GC, Agilent 7890B) featuring a thermal conductivity (TCD) and a flame ionization (FID) detector. At the end of each experiment, the liquid products were collected, and subsequently, quantified with a high-performance liquid chromatograph (HPLC, Shimadzu Prominence) and a liquid GC (L-GC, Shimadzu 2010 plus). Each experiment was carried out three times and the reported FEs represent the average value with the corresponding standard deviation of the different runs. Cyclic voltammetry (CV) scans were conducted in a potential range from −1.3 V to 1 V *vs.* RHE, whereby the anodic scan was performed first, followed by the cathodic sweep with a scan rate of 1.75 mV s^−1^. Before each CV experiment, the samples were reduced at −2.8 V *vs.* RHE (equals a current density of −500 mA cm^−2^) for 10 min.

### X-ray absorption spectroscopy

*Operando* QXAFS measurements were carried out at the Cu K-edge (8797 eV) and the Zn K-edge (9659 eV) in fluorescence mode using a PIPS detector. For XAS measurements, a home-build gas-fed cell was used that was identical to the reactor used for the selectivity studies, but featured a Kapton window for XAS data acquisition.^8^ A BioLogic (SP-300) potentiostat was used to control the potential. The measurements were conducted at the SuperXAS – X10DA beamline of the SLS synchrotron facility at the Paul Scherrer Institute (Switzerland). A fast moving channel-cut Si(111) monochromator was used to achieve a time resolution of one spectrum per second (1 Hz). The XANES and EXAFS spectra were extracted and analyzed by using Athena (Demeter Package^33^) and home-built *Wolfram Mathematica* scripts. A detailed description of the XAS data acquisition and analysis is given in the ESI† (Note 4). In this study, we complemented the conventional XAS data analysis with a novel machine learning-based (artificial neural network) approach for the EXAFS data interpretation, which is discussed in detail below.

### Surface-enhanced Raman spectroscopy

*Operando* SERS was used to analyze the presence of surface adsorbates and to interrogate the surface structure of the catalyst. For the SERS measurements, we used a home-built gas-fed cell with an opening at the electrolyte side for the immersion of the Raman objective close to the catalyst surface. A Raman spectrometer (Reinshaw, InVia Reflex) coupled with an optical microscope (Leica Microsystems, DN2500M) together with a motorized stage for sample tracking (Reinshaw, MS300 encoded stage) was used. A BioLogic (SP-300) potentiostat was used to control the potential in operando SERS measurements.

## 3 Results

### A) STEM measurements for as-prepared and reacted catalysts

**Fig. 1:**
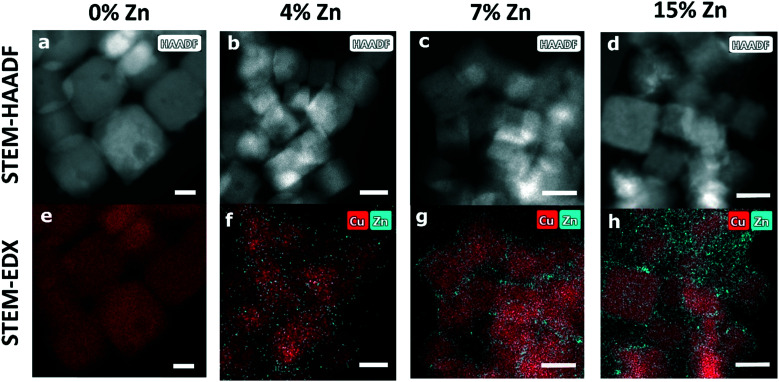
(a–d) STEM-HAADF and (e–h) STEM-EDX images in as-prepared state for (a and e) pure Cu_2_O NCs and bimetallic CuZn NCs with (b and f) 4% Zn, (c and g) 7% Zn and (d and h) 15% Zn. The white scale bars indicate a length of 20 nm.

STEM-HAADF and STEM-EDX data (Fig. 1, Table S1†) confirm that the samples in as-prepared state have narrow particle size and shape distributions. The Cu species form well-defined Cu_2_O nanocubes with an edge length of *ca.* 25 nm. The analysis of the corresponding STEM-EDX maps (Fig. S1 in ESI†) shows that the Cu_2_O NCs consists of 63% Cu and 37% O, which coincides with the Cu to O ratio in bulk Cu_2_O. The Zn-decorated samples in as-prepared state exhibit an evenly distributed Zn shell around the cubic Cu_2_O structures, with a thickness of *ca.* 2 nm for the CuZn catalysts with higher Zn loadings (7% and 15% Zn) and of less than 1 nm for the CuZn sample with a Zn loading of 4%.

We emphasize that the well-defined cubic shape of our pre-catalysts is at least partially lost under reaction conditions and the distribution of Zn species also experiences significant transformations (Fig. S2, Table S1†). In the STEM images of the samples that were exposed to CO_2_RR at a current density of −500 mA cm^−2^ for 1 hour, we observe morphological changes in all samples, suggesting an agglomeration of the Cu_2_O NCs. For the Zn-decorated samples with a Zn loading of 4% and 7%, the STEM-EDX maps reveal that after the harsh reaction conditions employed the Zn species form islands of approx. 8 nm and accumulate in what we hypothesize are cavities emerging in the deformed Cu NCs, while for the bimetallic sample containing 15% Zn a more uniform redispersion of Zn is observed.

### B) CO_2_RR selectivity for pristine and Zn-decorated Cu_2_O NCs at high current densities

**Fig. 2:**
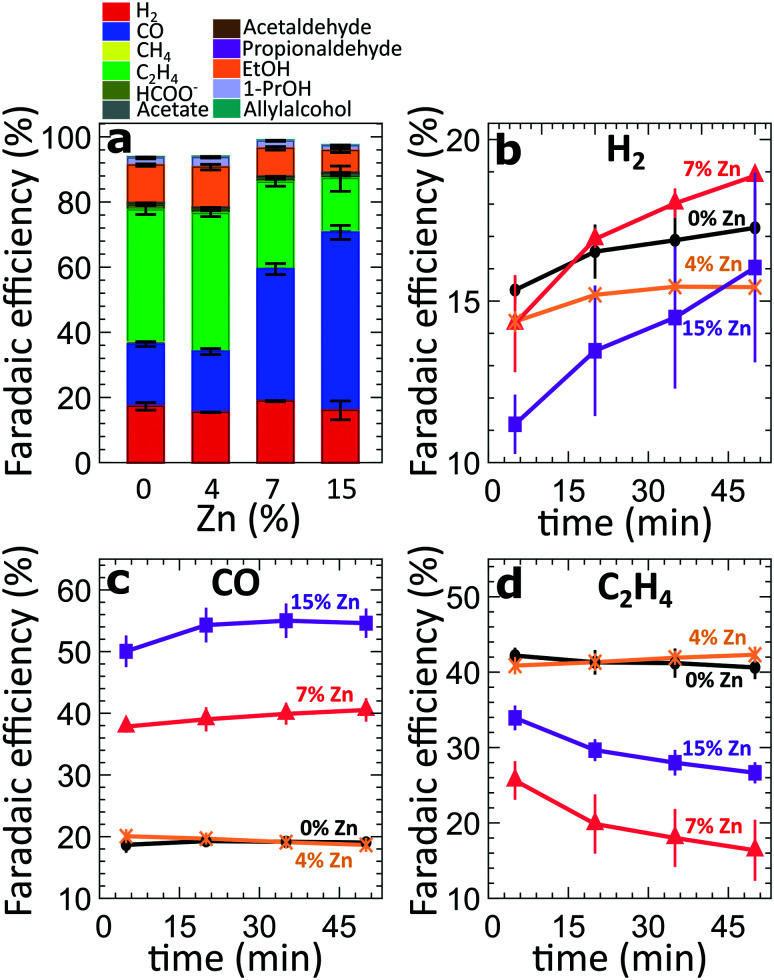
(a) FEs of all catalysts after 50 minutes of CO_2_RR at −500 mA cm^−2^. (b–d) Time-dependent changes of the FEs of all gas products during the first 50 minutes of CO_2_RR at −500 mA cm^−2^.

For the four different catalytic systems under investigation (derived from the pure and Zn-decorated Cu_2_O NCs with 4%, 7% and 15% Zn in as-prepared state), we compared the product distribution after applying CO_2_RR conditions for 60 minutes at high current densities, namely −500 mA cm^−2^ (Fig. 2a) and −250 mA cm^−2^ (Fig. S3†). In agreement with our previous study on Cu_2_O-derived catalysts,^8,10,25^ the pure Cu_2_O-derived catalysts show excellent activity and selectivity for the conversion of CO_2_ to hydrocarbons and oxygenates, with a relatively low FE for the parasitic HER (FE = *ca.* 13–16%), Fig. 2. The main CO_2_RR products that we detect at −500 mA cm^−2^ are ethylene (FE = 41%), CO (FE = 22%) and ethanol (FE = 12%). The introduction of Zn, however, results in significant changes in the catalyst selectivity. The most obvious change is a systematic increase of the CO FE with increasing Zn loading (reaching 18–53% FE). At the same time, the FEs of the main C_2_ products (ethylene and ethanol) at −500 mA cm^−2^ are similar (slightly larger) for the sample with the lowest Zn loading (FE = 55% for the 4% Zn) with respect to the FEs observed for pure Cu_2_O catalysts (FE = 52%). For the sample with the 4% Zn loading, we also see increased FEs of minor C_3_ products (1-propanol, allylalcohol and propionaldehyde), Fig. S4†, with respect to those for pure Cu_2_O NCs. A further increase in the Zn loading results in a decrease of the FEs of all of these products. The FE for ethylene decreases down to 16%, while the FE for ethanol decreases to 3.7% for the sample with 15% Zn loading, indicating less C–C coupling for more Zn-rich samples. For all samples, the Faradaic efficiency of the HER remains at a similarly low level. The selectivity trends observed in experiments at a current density of −250 mA cm^−2^ are qualitatively similar to those at −500 mA cm^−2^. However, in the case of −250 mA cm^−2^, the increase of the FEs for ethylene and ethanol was not observed for the sample with 4% Zn loading. The absolute values of the FEs also depend on the applied current density (Fig. S4 and S5†). We attribute the differences in the product selectivity between the selected current densities primarily to the potential dependence of the CO_2_RR. Nevertheless, as we show below, the current density values also affect the kinetics of the catalyst reduction and its structural transformations, resulting in different time-dependencies of the catalytic properties.

To take a closer look at the time-dependence of the catalytic properties, we have compared the FEs for the gaseous CO_2_RR products during the initial 50 minutes under CO_2_RR conditions at −500 mA cm^−2^ (Fig. 2b–d). For all samples, we observe an increase in hydrogen production over time (Fig. 2b). This increase is especially pronounced for the samples with a higher Zn loading. All samples decorated with Zn produce lower amounts of H_2_ immediately after the onset of CO_2_RR conditions in comparison to the pure Cu_2_O system. These findings might imply that the presence of Zn could suppress the parasitic HER. Nevertheless, with time the hydrogen production increases, and reaches a similar level as observed for the pure Cu_2_O sample, indicating that significant changes in the catalyst structure and/or morphology take place within this time. The increase in hydrogen production for the sample with 15% Zn loading, for instance, is paralleled by an increase of CO formation within the first 20 minutes, and a drop in ethylene FE by *ca.* 10%. A similar decrease in the ethylene production can also be observed for the sample with 7% Zn loading, which exhibits an increase in CO formation and a significant boost in H_2_ production with time. Interestingly, for samples with lower Zn loadings and for the pure Cu_2_O sample, no significant time-dependent changes in CO and ethylene production can be observed. We also note that the analogous time-dependent study of the gaseous reaction products at −250 mA cm^−2^ (Fig. S6†) reveals similar trends for CO and C_2_H_4_, but, except for the sample with 7% Zn loading, no significant increase in H_2_ production was observed there.

### C) QXAFS analysis at different current densities

**Fig. 3:**
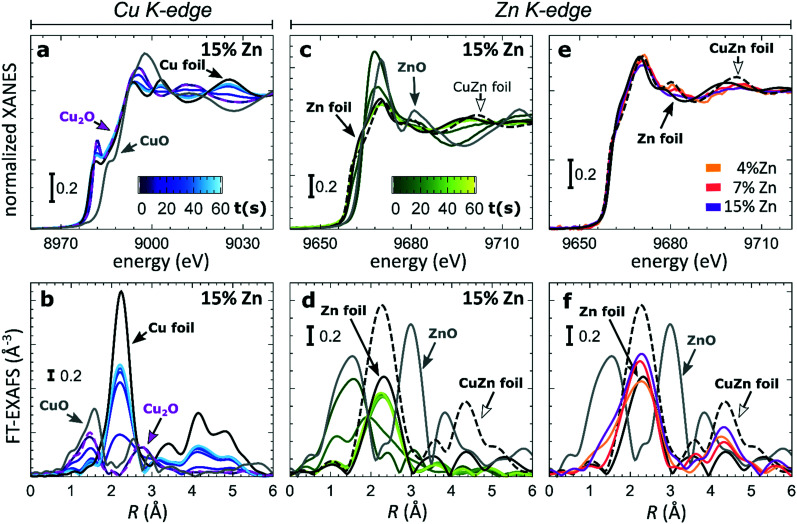
The evolution of XANES and Fourier-transformed (FT) EXAFS spectra (a and b) at the Cu K-edge, (c and d) and the Zn K-edge during the first minute of CO_2_RR at −500 mA cm^−2^. (e and f) The final XANES and FT-EXAFS spectra at the Zn K-edge after 1 hour of CO_2_RR.

To understand the origin of the observed differences between samples with different Zn loadings, and the discussed time-dependent selectivity changes, we used *operando* QXAFS to identify the local structure and composition in all of our catalysts and to follow their evolution. The representative Cu K-edge and Zn K-edge XANES and Fourier-transformed (FT) EXAFS spectra collected for the sample with 15% Zn loading under CO_2_RR conditions at −500 mA cm^−2^ within the first minute are shown in Fig. 3a–d, while the spectra for all other samples, including the raw EXAFS data, are shown in Fig. S7–S10.† Furthermore, a comparison of the Cu K-edge and Zn K-edge spectra measured after 1 minute and after 1 hour of CO_2_RR is shown in Fig. S8–S10,† respectively. Fig. 3e and f compare the Zn K-edge XANES and FT-EXAFS spectra collected after 1 hour under CO_2_RR conditions for samples with different Zn loadings. Our data show that the main changes in the catalyst's structure and composition at such high current density take place within the initial 60 seconds under applied current, motivating the need for the high time-resolution of the QXAFS method.

At the Cu K-edge, by comparing the XANES spectra of our catalysts with those of reference compounds (Cu foil and Cu_2_O, Fig. 2a), we conclude that most of the Cu(i) species in the as-prepared samples are quickly reduced to their metallic state under CO_2_RR conditions. These changes in XANES spectra are paralleled by the changes that we observe in FT-EXAFS data (Fig. 2b). Here, the peak at *ca.* 1.4 Å (phase uncorrected), which corresponds to Cu–O bonds in a Cu_2_O structure, disappears within the first minute under applied current, while the peak at *ca.* 2.2 Å, which arises from Cu–Cu bonds, increases. In all samples, the final state of the Cu species exhibits a peak in the FT-EXAFS data at the same distances as seen in metallic Cu, however, with a reduced amplitude (Fig. S9†). We attribute this reduced signal amplitude to the structural disorder in our catalysts, and/or the presence of small amounts of remaining oxide species. For similar catalysts, we observed a similar effect in the Cu K-edge EXAFS data in our previous study.^25^

Considering that the Cu to Zn ratio is large in all samples, it is not surprising that from the Cu perspective we do not see evidence of Cu and Zn interactions. The Zn K-edge is expected to be much more informative in this case. In the XANES Zn K-edge spectra, we observe that Zn is first present in an oxidized state, yielding a XANES spectrum with features resembling those visible in the reference spectrum for ZnO. However, the position and intensities of the Zn K-edge XANES features differ clearly from those in the bulk oxide reference, suggesting a strongly disordered structure. Under CO_2_RR conditions, XANES and EXAFS spectra change significantly, which indicates the reduction of Zn species. For example, for the Cu_2_O NCs decorated with 15% Zn (Fig. 2d), the initial peak in the Zn K-edge FT-EXAFS data at *ca.* 1.6 Å associated with Zn–O bonds disappears and over time a peak arises at *ca.* 2.3 Å, which we attribute to Zn–M bonds (M = Zn or Cu). We note that due to the broad shape and low intensity of this peak, it appears similar to the main FT-EXAFS feature in the reference data for metallic Zn. However, in our catalyst, this peak is shifted toward shorter interatomic distances, thus suggesting the presence of short Zn–M bonds, typical for an fcc-type CuZn alloy (brass). The Zn K-edge FT-EXAFS spectra are also clearly different for samples with different Zn loadings. In particular, in the final state for the samples with 4% and 7% of Zn, the contributions of distant coordination shells (peaks between 3–5 Å) resemble strongly those present in an fcc-type brass alloy. Meanwhile, for the sample with the highest Zn loading of 15%, the contributions of distant coordination shells are strongly suppressed. Based on this visual examination of Zn K-edge XANES and EXAFS data, we conclude that our samples feature a mixture of oxidized Zn species and regions of Cu-rich (fcc-type) and Zn-rich (non-fcc type) CuZn alloys, where the weights of the different species change with time under the applied current and depend also on the initial Cu to Zn ratio in the as-prepared samples.

**Fig. 4:**
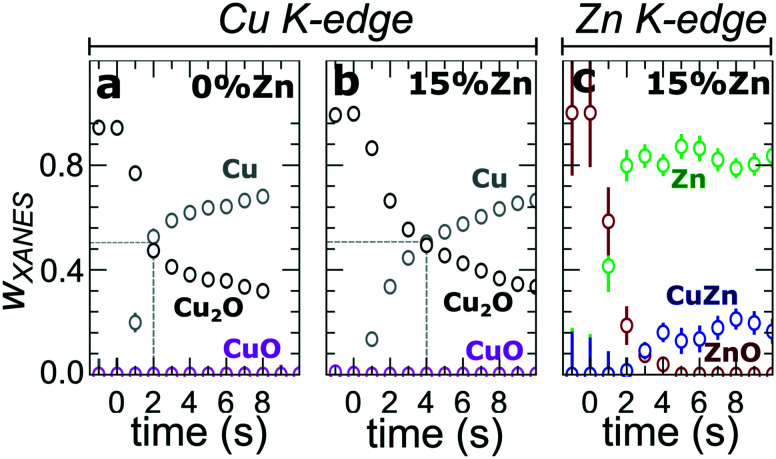
Time-dependent changes in the weight of different Cu and Zn species, as derived from the LCA of XANES data collected during the CO_2_RR at −500 mA cm^−2^ at (a and b) Cu K-edge and (c) Zn K-edge for the pure Cu_2_O NCs (a) and for the CuZn NCs (b) with a 15% Zn loading.

In our first attempt for quantification of the different Cu and Zn species present in each sample, we performed linear combination analysis (LCA) of XANES spectra (Fig. S11†). The LCA was done by expressing the XANES spectra of our catalysts as a linear combination of reference spectra collected for bulk oxides (Cu_2_O and CuO, ZnO) and metal foils (Cu foil, Zn foil, and brass foil (Cu_70_Zn_30_ alloy)). For Cu K-edge data, this approach works well (Fig. S11a†). The application of the LCA method for the interpretation of Zn K-edge XANES, in turn, requires more caution. We note that the local structure of all species that we expect to be present in our samples (oxidized Zn, Cu-rich and Zn-rich metallic phases) deviates significantly from the local structure of our reference materials (wurtzite-type ZnO, Cu_70_Zn_30_ brass foil, and metallic Zn foil). As a result, the agreement between our LCA-XANES fits and experimental data is not ideal (Fig. S11b and c†). The tentative results, summarized in Fig. 4, suggest that both, Cu and Zn are rapidly reduced at a current density of −500 mA cm^−2^. Zn(ii) species are reduced within the first 4 seconds under applied current, which is faster than the observed reduction of Cu(i) (Fig. 4a and b). In particular, we observe that the presence of Zn strongly affects the reducibility of copper (Fig. S12†). In pure Cu_2_O NCs more than half of the copper oxide species is reduced after 2 seconds, whereas in the presence of Zn a similar degree of Cu reduction is reached only after 4 seconds. We also observe that after the rapid initial reduction of copper, small amounts of Cu(i) species still remain in our samples and that the ratio of Cu(0) : Cu(i) was found to be stable after 1 hour of CO_2_RR (Fig. S13†). Interestingly, we observe that with increasing Zn content, the amount of residual Cu(i) oxide at highly negative potentials is also increased (*e.g.*, for the pure Cu_2_O samples it is 4%, while for the sample with 15% Zn loading it reaches 10%). This indicates that the presence of Zn could help to stabilize the Cu(i) species.

Furthermore, LCA-XANES analysis at the Zn K-edge suggests that after the fast reduction of Zn(ii) to Zn(0), a more gradual transformation of metallic Zn toward Cu_*x*_Zn_*y*_ alloys takes place. Here, the LCA-XANES fitting allows us to track the ratios of the different reduced Zn species to some extent, but misidentification of the phases can occur. For example, Zn-rich phases could face considerable shortening of the Zn–M distances due to alloying (as implied by the visual examination of Zn K-edge EXAFS data), potentially affecting the Zn K-edge XANES features. Moreover, the formation of Cu-rich alloy phases with Cu contents much higher than in the common Cu_70_Zn_30_ brass can increase the complexity of the system, but this phenomenon is hard to identify with the LCA-XANES analysis, due to the lack of available reference spectra for different Cu-rich alloys. We will address this issue below, by relying on EXAFS data and applying a machine learning-based method. Overall, our LCA-XANES results suggest that the final structure is different for samples with different Zn loadings. It can be seen that for samples with lower Zn loadings, higher ratios of fcc-type alloys are formed, while in the Zn-rich catalysts, significant amounts of species with a local environment similar to that in metallic Zn are observed.

For comparison, the LCA-XANES results obtained at a much lower current density (−10 mA cm^−2^), are shown in Fig. S13a–d.† The lower current density results in a much slower reduction of the species. Furthermore, in the final state of our samples, a higher contribution of alloy species is seen from Zn K-edge LCA-XANES data when reducing them at −500 mA cm^−2^ as compared to the alloy contribution when applying −10 mA cm^−2^. This effect, however, seems to be less pronounced for samples with higher Zn loadings.

### D) Neural network approach for deciphering EXAFS spectra arising from heterogeneous composites

To overcome the limitations of the LCA-XANES analysis, we have developed a new approach for the quantification of different alloy phases, based on EXAFS data and machine learning. This method is built upon ideas from our previous works, where an artificial neural network method was used to “invert” EXAFS spectra for metals and oxides, and directly extract the radial distribution function of neighboring atoms of different types.^19,29,34–36^ We rely on the observation that for a mixture of different species, the total EXAFS spectrum *χ*(*k*) can be expressed as a sum over partial contributions of different species 
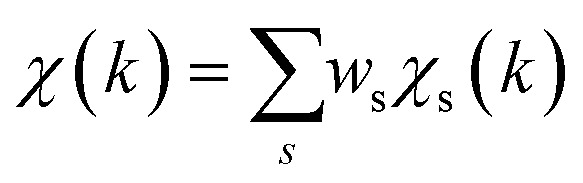
 where *w*_s_ is the concentration of the species, and *χ*_s_(*k*) is the partial spectrum for the pure species, which is linked to its structure. For example, in the single scattering approximation, *χ*_s_(*k*) can be directly linked to the partial radial distribution function (RDF) *g*(*R*):1
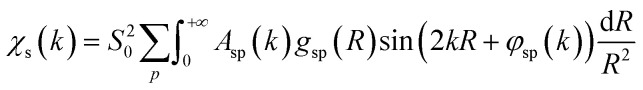
Here, the summation is carried out over distinct bonds (more precisely – photoelectron scattering paths). *φ*_sp_ and *A*_sp_ are the scattering phase and amplitude terms that can be calculated *ab initio* for *s*-th species and *p*-th path with, *e.g.*, the FEFF code,^37^*R* is the half-length of the path, therefore related to the interatomic distance between neighbouring atoms, and *S*^2^_0_ is the amplitude reduction factor originating from many-electronic effects. In our previous works,^19,29,35,36^ we have shown that *g*_sp_(*R*) functions can be extracted from EXAFS spectra using machine learning. In this process, we first construct large sets of theoretical relevant structure models. For each model, we calculate the corresponding *g*_sp_(*R*) functions (parametrized as a histogram of interatomic distances between atoms of given types) as well as the corresponding *χ*_s_(*k*) spectrum. Next, we create a new set of spectra, by generating linear combinations of theoretical *χ*_s_(*k*) spectra 
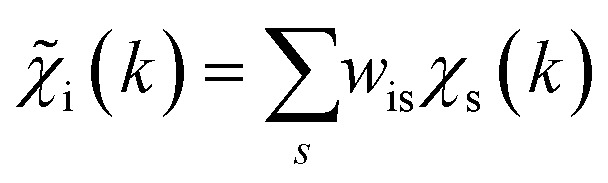
 with arbitrary weights *w*_s_, and the corresponding set of mixed RDFs 
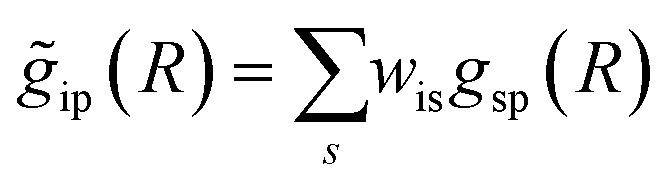
. An artificial neural network (NN) is then constructed and trained using the generated theoretical data set to map the relationship between 
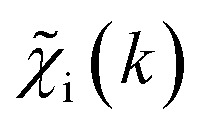
 and *g̃*_ip_(*R*). The *g̃*_ip_(*R*) vectors for different *p*-values were concatenated, forming one large output vector. Then, during the training, the parameters of the NN nodes were optimized to ensure that the NN output matches the known true value of *g̃*_ip_(*R*) when the training spectrum 
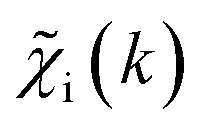
 was provided as input. After the training was completed, the NN parameters were fixed, and an experimental EXAFS spectrum could be provided as an input, giving us an output vector corresponding to *w*_s_*g*_sp_(*R*) for all non-equivalent bonds *p*. We have demonstrated that such an approach can successfully retrieve partial Pd–Au and Pd–Pd RDFs in PdAu nanoparticles,^34^ and extract partial Zn–O and Zn–M RDFs in oxidized CuZn catalysts.^19^ In both these cases, the success of the NN-EXAFS method for RDF extraction was ensured by a sufficient spectral contrast between Pd–Au/Pd–Pd and Zn–O/Zn–M contributions. However, we highlight that such spectral contrast is not necessarily required for the NN method to work. Indeed, the *g*_sp_(*R*) functions that we aim to obtain from EXAFS data are not arbitrary functions of *R*, but are constrained by our training procedure to resemble the theoretical *g*_ip_(*R*) functions used for NN training. If all the considered species have sufficiently different structures (each resulting in sufficiently different EXAFS spectra for pure compounds), the mixture of, *e.g.*, two species will produce the total EXAFS spectrum that is not possible to obtain by mixing EXAFS spectra of any other two species, even if these species contain atoms of the same type. This allows us to reformulate our NN-EXAFS approach as follows. After calculating theoretical *χ*_s_(*k*) and *g*_sp_(*R*) for pure compounds, we, as done previously, mix the *χ*_s_(*k*) with arbitrary weights and use the obtained 
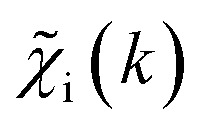
 as inputs for our NN. The difference is that now we request the NN output to be a concatenation of 
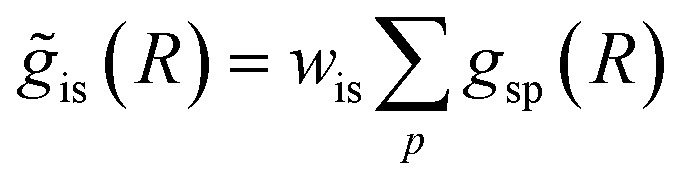
, where the summation is carried out over all distinct bonds present in the *s*-th species. Using these pairs of 
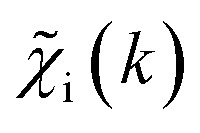
 and *g̃*_is_(*R*), we again perform the NN training, and then supply the experimental EXAFS spectrum, to obtain structural information on the sample of interest. Thus, if in our previous work,^19^ the NN output was the concatenated partial RDFs of O and M atoms around the absorbing Zn (each averaged over all Zn-containing species in the sample), in our new approach, the NN output is the concatenated total RDFs for three spectroscopically distinct phases: oxide, fcc-type metal, and non-fcc type metal. Each RDF is weighted with the concentration of the corresponding species in the mixture yielded from the analyzed EXAFS spectrum.

With this key distinction in mind, the further NN training and validation is carried out analogously as in our previous work.^19^ The details of the NN implementation are given in ESI† (Note S5). In particular, for the generation of theoretical training structures to model the effect of structural disorder, we relied on molecular dynamics and Monte Carlo simulations using simple empirical force field models. In addition to the potentials for metallic Cu, and Cu- and Zn-oxides, tested and validated in our previous work,^19^ this time we also included the structure models for metallic Zn in the training data set, which were created by using a modified embedded atom force field model from ref. 34. As shown in Fig. S14,† the EXAFS spectra calculated with this force field are in excellent agreement with experimental data. This makes this force field model well-suited for training ML routines for the interpretation of EXAFS data in Zn-rich systems.

As in our previous works,^35^ we validated the accuracy of the designed NN using experimental spectra for pure well-defined reference materials (oxides, metals, and alloys). In Fig. S15a and b,† we demonstrate that RDFs extracted by NN match well those independently obtained from the same EXAFS data by reverse Monte Carlo simulations^38–40^ (which can be used for the analysis of pure bulk materials, but not for mixtures).

Once the RDFs are obtained by NN-EXAFS methods, the quantification of the concentration of each of the phases in the mixture can be performed by integrating the obtained RDFs:2
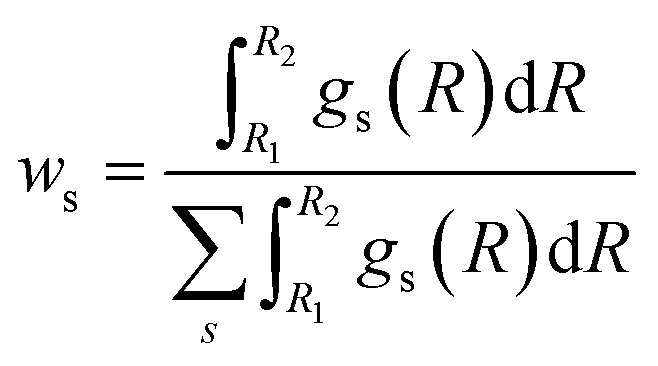
The obtained *w*_s_ values can be directly contrasted with those obtained from LCA-XANES. At the same time, the NN-EXAFS is more robust, since it does not require the sample to have the same local structure as that in the very limited number of experimentally measured reference compounds. Moreover, since our NN-EXAFS approach provides not only *w*_s_ values, but the entire RDFs, much more information can be obtained. For example, the obtained RDFs can be immediately used to identify shifts in interatomic distances due to alloying/dealloying.

The accuracy of the *w*_s_ values yielded by NN-EXAFS is tested in Fig. S15c–e.† Here, we first constructed artificial linear combinations of experimental Zn K-edge spectra for ZnO, Zn foil, and a CuZn (brass) foil, and then used them as input for the NN from which we obtained corresponding RDFs for oxide, non-fcc and fcc phases. Finally, we calculated the weights of different phases by using eqn (2). The obtained *w*_s_ values are in good agreement with the known true values, giving us confidence in the accuracy of the proposed approach. Examples of RDFs, reconstructed for these artificial mixtures, are shown in Fig. S16.†

### E) Application of NN-EXAFS analysis to QXAFS data

**Fig. 5:**
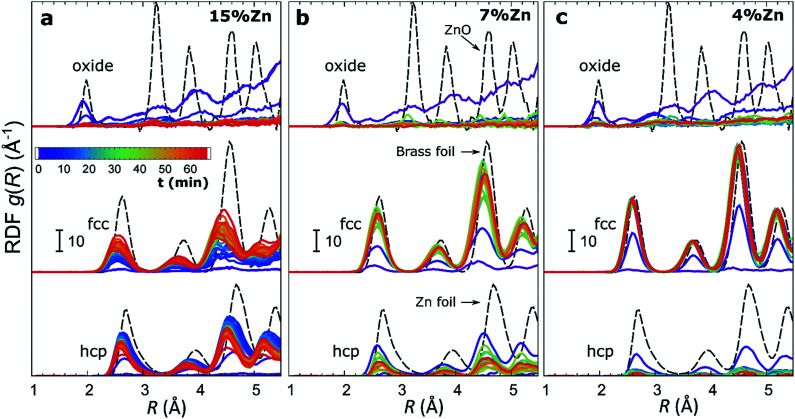
Time-dependent evolution of radial distribution functions obtained by the NN-EXAFS analysis from operando Zn K-edge EXAFS data for bimetallic catalysts with (a) 15%, (b) 7% and (c) 4% Zn content. Partial RDFs corresponding to an oxide phase, a metallic phase with fcc-type structure and a metallic phase with non-fcc type structure are shown. RDFs are shifted vertically for clarity.

**Fig. 6:**
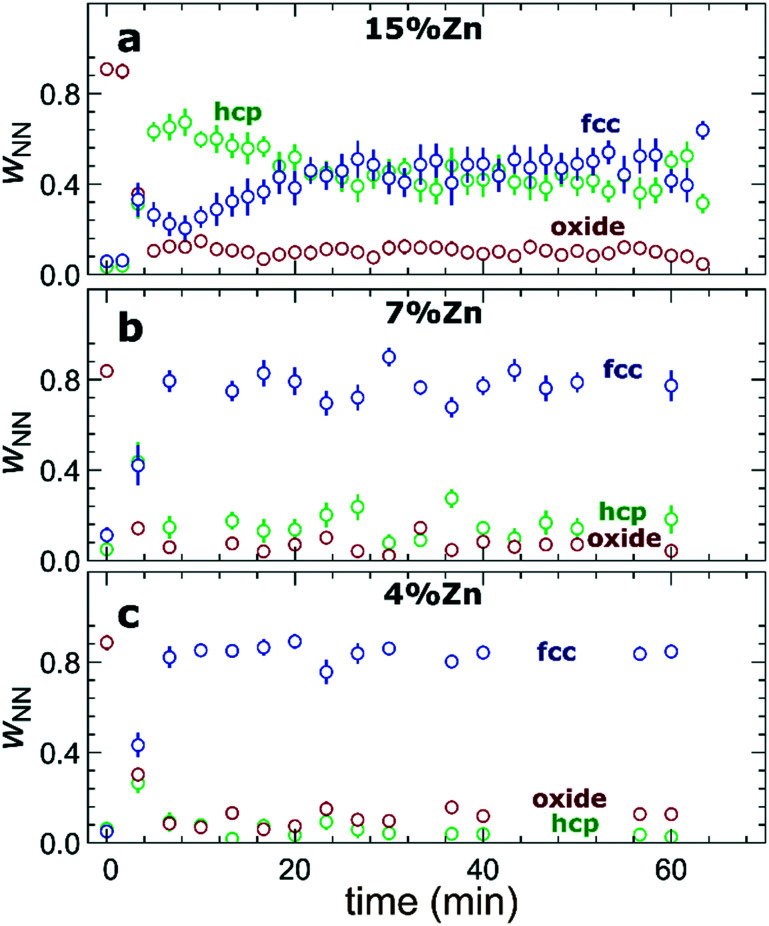
Evolution of the relative concentrations of oxide, fcc-like and hcp-like phases, as obtained by the NN from *operando* Zn K-edge EXAFS data for bimetallic catalysts with a Zn loading of (a) 15%, (b) 7% and (c) 4% during CO_2_RR at a current density of −500 mA cm^−2^.

The RDFs obtained by the NN-EXAFS method from experimental *operando* Zn K-edge QXAFS data at −500 mA cm^−2^ for Cu_2_O NCs decorated with 15, 7 and 4% Zn, respectively, are compared in Fig. 5a–c (and Fig. S17a–c† for −10 mA cm^−2^). In agreement with the results of the visual examination of the XANES and EXAFS data, and with the results of the LCA-XANES analysis (Fig. 4a), we see that in the as-prepared samples the Zn species are completely oxidized, and only the contributions of an oxide phase are detected. However, the broad, smeared-out peaks of the RDF suggest that the structure of the oxide is very different from the bulk ZnO structure, and that the local environment around the cationic Zn species is disordered and amorphous. Upon exposure to CO_2_RR conditions at −500 mA cm^−2^, the zinc oxide is rapidly reduced and replaced by the fcc-like (typical for Cu-rich CuZn alloys) and non-fcc type (typical for metallic Zn and Zn-rich CuZn alloys) metallic phases. The evolution of oxide, fcc-alloy and non-fcc alloy fractions with time is compared in Fig. 6 for the samples with different Zn loadings. The contribution of the fcc-like phase is continuously increasing with time, while for the hcp-like phase the maximum contribution under applied current is reached after 5–10 minutes. After that, its contribution gradually decreases. This indicates that the alloying of Cu and Zn takes place and an increasingly Cu-rich alloy with an fcc-type structure is formed over time. Another factor, on which the contribution of the non-fcc phase strongly depends, is the initial Cu to Zn ratio. For the sample with 15% Zn loading, the contribution of the non-fcc phase is the highest and accounts for up to 70% of all Zn species after 10 min of CO_2_RR. After a longer exposure to reaction conditions, the contribution of the alloy phase gradually decreases, reaching a state of saturation after *ca.* 30–40 min. At this point, the contributions of the fcc-phase and the non-fcc phase are nearly equal in this sample. For samples with lower Zn loadings, the contribution of the fcc-type phase systematically decreases. For the samples with a lower Zn loading, the contribution of non-fcc Zn species is substantially less pronounced, and already after 20 min *ca.* 80% of all Zn species are present in the form of an fcc-alloy. Moreover, we can gain information from the positions of the RDF peaks, which reflect the interactions between Cu and Zn. From the observation that even in Zn-rich non-fcc phases all the interatomic distances are noticeably shorter than in the metallic Zn foil, we can conclude that the Zn lattice spacing decreases due to alloying. We note that the Zn–M distances in non-fcc phases continue to decrease with time, suggesting an increasing enrichment of this phase with Cu species as the Cu–Zn distances are shorter than Zn–Zn distances. The interatomic distances in the fcc-type alloy phase are similarly shorter than the Zn–M distances in typical fcc-type Cu_70_Zn_30_ brass, and suggest that the alloy formed in our catalysts is more Cu-rich (note here that the lattice constant of brass is by *ca.* 2.5% larger than the lattice constant of pure Cu (ref. 41)). Interestingly, for the samples with the lowest (4%) and highest (15%) Zn loadings, the final state of the catalyst does not significantly depend on the value of the applied current density. For the respective samples, a final concentration of different phases at −10 mA cm^−2^, similar to that at −500 mA cm^−2^, is reached after 1 hour of CO_2_RR (Fig. S18†). However, at −10 mA cm^−2^ the alloying process is clearly slower. In contrast, for the intermediate Zn loading (7%), the selected current density indeed affects the final catalyst structure. In particular, the sample treated at −500 mA cm^−2^ showed a noticeably higher final concentration of the Cu-rich fcc-type CuZn alloy (*ca.* 80%) as compared to the sample treated at −10 mA cm^−2^ (*ca.* 60%).

### F) Potential-dependent QXAFS study

**Fig. 7:**
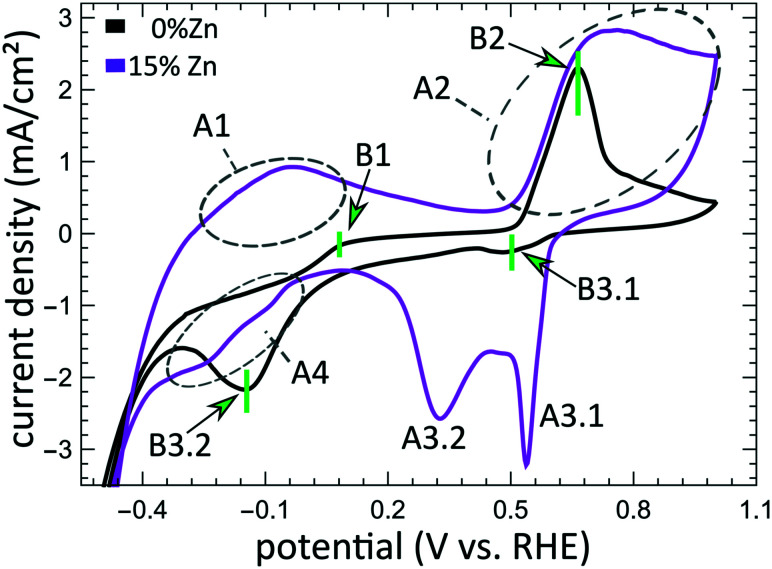
Cyclic voltammogram for the Cu_2_O and bimetallic Zn/Cu_2_O catalysts with different Zn loadings. The current density is plotted as a function of the potential. The CV peaks for the bimetallic CuZn samples are labelled as A1, A2, A3.1, A3.2, and A4, while the CV peaks appearing in the pure Cu_2_O catalyst are denoted as B1, B2, B3.1, and B3.2.

**Fig. 8:**
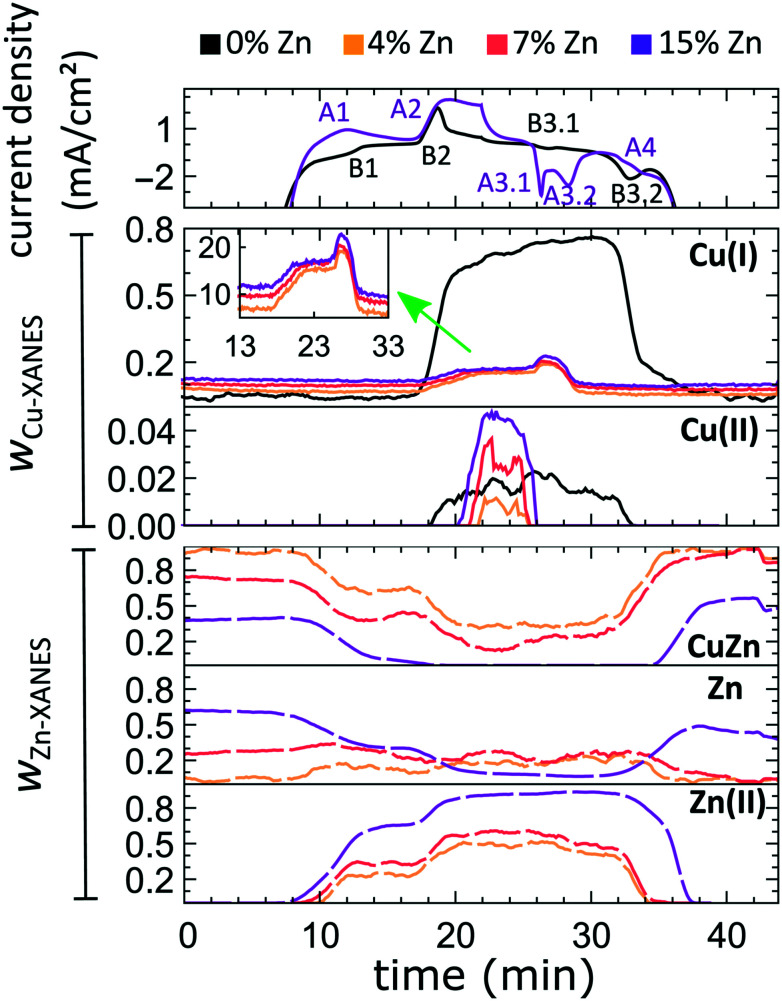
Time-dependent changes in the current density during the CV scan, and the corresponding changes in the concentrations of Cu(i), Cu(ii), brass-like Zn and metallic Zn-like species with the current density plotted as obtained by the LCA of Cu K-edge and Zn K-edge XANES data.

The aforementioned dependency of the reduction kinetics of Cu on the presence of Zn species is intriguing. To better understand the redox trends in our oxide-derived Zn/Cu_2_O catalysts and their dependency on the Cu to Zn ratio, we used QXAFS. Thus, we compared the evolution of the pure Cu_2_O NCs and the Zn/Cu_2_O NCs with different compositions during cyclic voltammetry (CV) scans. The recorded CVs for the pure Cu sample and the sample with 15% Zn loading are displayed in Fig. 7 and 8a. The respective CVs for 4% and 7% Zn-decorated samples, which are qualitatively similar to the CV of the 15% Zn-decorated sample, can be found in the ESI† (Fig. S19 and S20). Exemplary Cu and Zn K-edge XANES spectra for each sample during the anodic scan of the CV are shown in Fig. S21–S24.† Before the CV scans, the samples were kept at a cathodic potential (−2.8 V *vs.* RHE) for 10 min. The CV sweeps were started at a cathodic potential of −1.3 V *vs.* RHE, scanned until an upper potential of 1 V *vs.* RHE, and then cycled back to the starting potential. The scan rate of 1.75 mV s^−1^ was chosen to match the time resolution of our QXAFS measurements. The main features of the CV for Cu_2_O catalyst are marked in Fig. 7 as B1, B2, B3.1, and B3.2, and correspond to the CV peaks visible at *ca.* 0.7 V in the anodic scan (B1), at *ca.* 0.5 V (B2), at −0.5 V (B3.1), and at −0.2 V (B3.2) in the cathodic scan. The main features in the CVs for the bimetallic catalysts are marked as A1, A2, A3.1, A3.2, and A4. In particular, A1 corresponds to the region between −0.4 V and 0 V in the anodic scan, where two small split peaks are observed in the CVs for samples with 4% and 7% Zn loading, and one broad peak for the 15% Zn containing sample. A2 denotes the intense peak followed by a current plateau from 0.5 V until the upper vertex in the upward scan. Two sharp peaks at *ca.* 0.6 V and 0.3 V in the cathodic scan are indicated as A3.1 and A3.2, respectively. The broad feature between 0 V and −0.4 V in the cathodic scan is labeled as A4.

By using QXAFS in combination with LCA-XANES (Fig. 8) and the NN-EXAFS method (Fig. S25 and S26†), it is possible to directly correlate the current peaks in the CV scans to the chemical evolution of Cu and Zn species.^8^ In this case, the advantage of the LCA-XANES method is the higher achievable time-resolution, which is only limited by the QXAFS acquisition time (in our case – one second per spectrum). For the NN-EXAFS method, we averaged every 10 spectra to ensure sufficient signal to noise ratio in the EXAFS data, thus decreasing the time-resolution to 10 s per spectrum. However, more detailed information on the structural changes can be obtained from NN-EXAFS.

**Features A1 and B2/A2.** In the CV data, the anodic part of the CV scan is related to the oxidation of our catalyst. The detailed discussion and assessment of these CV features is presented in the ESI† (Note 6). However, we emphasize here that the feature A1 corresponds to the oxidation of Zn species, and that its intensity increases systematically with the Zn loading. Meanwhile, the features B2 and A2, which are the most pronounced features in the CVs for the pure Cu_2_O NCs, and for the Zn-decorated samples, respectively, correspond mostly to the oxidation of metallic Cu to the Cu(i) state, but the oxidation to the Cu(ii) state also contributes to this feature. Further, we note here that feature B1, which also appears during the anodic part of the CV scan, is only visible for the pure Cu_2_O sample, and cannot be correlated with any obvious changes in our XAS data averaged over the entire sample. This indicates that it may be related to processes restricted to the catalysts' surface and could be linked to changes in the interactions between the catalyst and adsorbates. In section 3G, we will identify this feature using the *operando* SERS method.

For the purposes of our work, the most important CV features are the peaks labelled as A3.1, B3.1, A3.2, B3.2, and A4, which can be observed during the cathodic part of the CV scan. These features are attributed to the reduction of our catalyst and can be unambiguously identified by XAS.

**Features B3.1 and A3.1** appear in the CV spectra together with the increase in the Cu(i) species concentration and the drop in the concentration of the Cu(ii) species, as visible from our LCA-XANES data (Fig. 8). Considering that according to the LCA-XANES data there is more Cu(ii) present in the bimetallic samples than in the pure Cu sample, feature A3.1 is more pronounced than the feature B3.1. One can also note that in the bimetallic samples the Cu(ii) species are reduced very quickly, while in monometallic Cu this process is more gradual. This observation can be attributed to the fact that monometallic Cu already contains much more Cu(i) species than the bimetallic samples, thus the further increase in Cu(i) concentration is not thermodynamically favored.

**Feature A3.2** is a broad, prominent peak in the CVs for the bimetallic samples at *ca.* 0.3 V. LCA-XANES clearly shows that this feature corresponds to the reduction of Cu(i) species to the metallic Cu state. We emphasize here that at this point in monometallic Cu samples, the contribution of Cu(i) is not noticeably decreasing in our bulk sensitive XAS data. As we will show later, the situation is different when surface-sensitive SERS measurements are employed.

**Feature B3.2** in the CVs of the monometallic Cu sample corresponds to the reduction of Cu(i) to Cu(0) and is thus analogous to feature A3.2. One should stress here that this feature in pure Cu_2_O NCs is shifted by almost 0.4 V to more negative potentials with respect to feature A3.2 in bimetallic samples. This difference shows the significant impact that the presence of Zn species can have on the reducibility of the Cu moieties in our catalysts.

Finally, feature A4 marks the reduction of Zn(ii) species. Similarly, as with the oxidation peaks, this feature is also broad and several smaller peaks can be resolved here, corresponding to the formation of metallic Zn and CuZn alloys with different Cu to Zn ratios. The structure of the reduced Zn species remains nearly unaffected by the intermittent oxidation cycle in all Zn-decorated samples regardless of their initial Zn content. From both LCA-XANES and NN-EXAFS results, it became clear that the samples with a low Zn loading exhibit similar amounts of the Cu-rich fcc-type phases after the transformation of zinc oxide back to reduced Zn species as before the oxidation. Similarly, in the sample with the highest Zn loading, after oxidation and reduction the contribution of the Zn-rich non-fcc phase remains to be significant. In this case, however, an increase by *ca.* 10 percentage points of the contribution of the fcc-type alloy phase can be detected, which could be attributed, in part, to the irreversible dissolution of Zn(ii) deriving from a non-fcc Zn state during the oxidation step, and, on the other hand, to the concurrent alloying with Cu during the repeated reduction.

### G) Surface-enhanced Raman spectroscopy

**Fig. 9:**
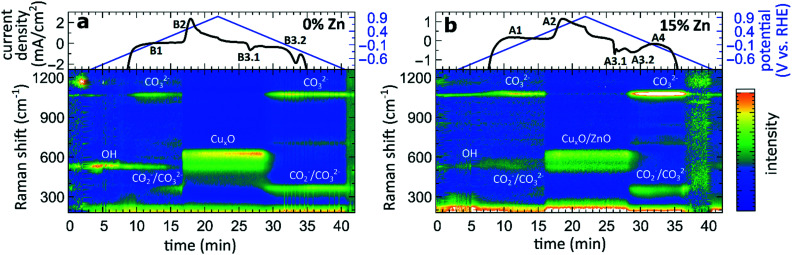
Time-dependent SERS band intensities during the CV scan, and the corresponding changes in the current density for (a) pure Cu_2_O NCs and for the (b) Zn/Cu_2_O catalyst with a 15% Zn loading.

To complement the sample-averaged bulk-sensitive information about the catalyst's composition and structure from the *operando* QXAFS analysis, we conducted time-resolved *operando* SERS measurements to highlight the role of surface species during the oxidation and reduction of the catalysts. Moreover, SERS is particularly sensitive to surface species on plasmonic materials such as nanostructured Cu.^32^ In Fig. 9, the SERS intensities of the pure Cu_2_O sample and the sample with the highest Zn loading (15% Zn) collected during a CV scan are shown in the range of 200 to 1250 cm^−1^. The data collected for the sample with 4% and 7% Zn loadings resemble the results for the 15% Zn sample and are depicted in Fig. S28.† Further, selected SERS spectra of all catalysts during open circuit potential, at −1.3 V, and during the CV can be found in the SI (Fig. S29†).

In the CO_2_RR-relevant potential region (below *ca.* −0.9 V), the SERS background intensity is significantly boosted due to the strong bubble formation during the CO_2_RR. However, the main characteristic bands, namely the restricted rotation band of adsorbed CO at 280 cm^−1^, the stretching band of adsorbed Cu–CO at 360 cm^−1^,^32^ and the vibration of adsorbed OH^−^ at 530 cm^−1^,^42^ are clearly visible in the corresponding spectra collected before and after the CV at −1.3 V (Fig. S29†). While only the OH^−^ band remains upon increasing the applied potential, another weak band at 1070 cm^−1^ is present at those negative potentials, whose intensity significantly increases at *ca.* −0.6 V. This band corresponds to the monodentate CO_3_^2−^ stretching mode.^43^ The increase of the CO_3_^2−^ band intensity coincides with the decrease of the intensity of the OH^−^ adsorbate.

Simultaneously, new bands with a Raman shift of *ca.* 360 cm^−1^ and 710 cm^−1^ appear at approx. 0 V. In previous works, the bands at 360 and 710 cm^−1^ were assigned to surface Cu carbonate species CO_3_^2−^ ^32,44^ or adsorbed carboxylate CO_2_^−^.^45,46^ An alternative interpretation was provided in a recent work, where the peak at 710 cm^−1^ was assigned to hydroxyl species.^47^ In those previous studies, however, these were only observed during the reduction cycle and related to the reduction of Cu_2_O to Cu. In our study, however, we observe them also before the Cu oxidation. One reason for that might be the 10-fold higher concentration of KHCO_3_ in our study, which could lead to the additional formation of a carbonate adsorbate layer before the oxidation of metallic Cu. These adsorbate bands are present in the SERS spectra of all catalysts and align well with the feature B1 observed in the CVs for the pure Cu_2_O sample (Fig. 7). For bimetallic samples, we observe from SERS a similar evolution of carbonate species. We suspect that an analogous peak in the CVs cannot be observed due to an overlap with the broad CV feature A1, which corresponds to the oxidation of Zn species in bimetallic catalysts.

As the potential reaches *ca.* 0.5 V, the Raman bands of the adsorbates are replaced by the characteristic bands of Cu_*x*_O at 410, 530 and 620 cm^−1^,^42,43^ and the SERS result thus matches well that obtained from XAS data interpretation (features A2 and B2). In all CuZn catalysts, ZnO bands, commonly appearing at 440 and 570 cm^−1^, could be overlapping with the Cu_*x*_O bands, and are therefore challenging to detect.

During the cathodic scan, the prominent SERS bands of Cu_*x*_O disappear at a potential of *ca.* 0.3 V. Strikingly, in contrast to XAS data, SERS shows that the reduction of Cu in monometallic and bimetallic catalysts takes place at the same potential value. At the same time, the prominent CV feature B3.2, which, according to XAS, we unambiguously attributed to the Cu reduction in monometallic Cu samples, is not reflected in the SERS data at all. Considering the different probing depths of the SERS and XAS methods, we attribute this difference to the fact that surface oxide species (probed by Raman spectroscopy) are reduced more easily than the bulk oxide species (whose contributions dominate the XAS data for strongly oxidized catalysts). After the reduction of the oxide surface species at 0.3 V, the SERS bands at *ca.* 360, 710 and 1070 cm^−1^ of the adsorbates start to appear again, while the OH^−^ band at a Raman shift of *ca.* 530 cm^−1^ is reappearing in the negative potentials of the CO_2_RR regime.

## 4 Discussion

In this study, the role of Zn in bimetallic Cu-based electrocatalysts was illustrated from different perspectives. We have shown that all our catalysts experience significant morphological, chemical, and structural changes when exposed to CO_2_RR working conditions. The NCs face transformations of their shapes, including the agglomeration and redistribution of Zn species as well as the formation of Zn islands. Further, the reduction of Zn and Cu species and the formation of CuZn alloys with different ratios of the constituent metals and different crystallographic structures (oxide, fcc and non-fcc) depend on the initial Cu to Zn ratio in the pre-catalyst and on the applied current density. Importantly, we have shown that at high current densities the evolution of the catalysts' structure takes place on at least two different time scales: the quick reduction of the Cu and Zn species, which is completed within seconds, followed by a slow alloying, which requires hours and is accompanied by catalyst restructuring processes. The kinetics of these processes depend on the catalyst composition, as well as on the current density under operation conditions. These findings provide a broad range of possibilities for rational tailoring the catalyst composition, structure, and ultimately, its functionality. In particular, as demonstrated in our study, the interplay between these parameters resulted both in differences in the catalytic selectivities for samples with different compositions, as well as in differences in the time-dependent evolution of their catalytic properties. Below, we discuss several aspects of the CO_2_RR process, where the role of Zn is found to be decisive.

### Promotion of CO selectivity

When used as an independent CO_2_RR catalyst, metallic Zn converts CO_2_ into CO as the main reaction product.^48,49^ Our findings from LCA-XANES and NN-EXAFS data show that large fractions of reduced Zn species with a local structure similar to that of hcp-type metallic Zn are mainly present in samples with high Zn loading under CO_2_RR conditions (Fig. 4, 6, S13 and S18†), which explains the observed trend in CO selectivity. Namely, the CO production is significantly higher for samples with high Zn loadings than for samples with low Zn loadings and pure Cu (Fig. 1a and S3†). We note here that it was shown previously that the selectivity of Zn toward CO increases with the size of Zn particles,^50^ thus the systematic increase of the FE for CO that we observe upon an increase in the Zn loading could also indicate that the effective sizes of the Zn-rich regions in our catalyst become larger. However, our post-mortem STEM-EDX analysis (Fig. S2†) suggests that this is not the case for our samples, and, in fact, we observe better dispersion of Zn in the Zn-rich samples than in the Zn-poor samples.

### Alloying/dealloying effects

The observed changes in the FEs for the gaseous reaction products (Fig. 1b–d) take place on the time scale of *ca.* 1 hour, which coincides with the characteristic time of alloying processes (Fig. 6, S13 and S18†) and is thus much slower than the characteristic time for Cu and Zn reduction (Fig. 3 and 4). This allows us to conclude that alloying of Cu with Zn plays a major role in determining the product selectivity for CuZn catalysts. In particular, we observed a correlation between the time-dependent transformation of the metallic Zn layer toward Cu_*x*_Zn_*y*_ alloys and the concomitant increase of hydrogen formation with time. Concurrently, the selectivity for ethylene decreases with time, especially for bimetallic catalysts with higher Zn loadings. Under CO_2_RR conditions at a current density of −500 mA cm^−2^, only for the sample with the lowest Zn loading (4%), we observed a selectivity for ethylene and ethanol as high as for the pure Cu_2_O sample. These observations allow us to conclude that only very Cu-rich CuZn alloys could be beneficial for the formation of multicarbon species. Consequently, the prolonged alloying results in increasingly Zn-rich alloys, and, hence, in a decrease of the FEs for multicarbon products. Backed by our earlier work, which was performed at low current densities,^13^ these findings indicate that the spillover effect expected in bimetallic catalysts, which leads to enhanced C–C coupling, only takes place at an unaltered catalyst surface with a uniformly spread metallic Zn shell or in the presence of relatively low amounts of Zn, where no extensive alloying takes place. We also note here that the alloying of Cu and Zn in these materials is reversible. This is shown in Fig. S24,† where the partial oxidation of Zn species results in shorter Zn–M bonds in the remaining metallic Zn component, signaling the presence of alloys with lower Zn content. According to the discussion above, these de-alloyed phases could potentially be better suited for the CO_2_ conversion to multicarbon products.

### Changes in Cu reduction kinetics: high current densities

Our LCA-XANES and NN-EXAFS results suggest that the oxidation state of the main active species (Cu) under CO_2_RR conditions is affected by the presence of Zn. In particular, we observed that the reducibility of Cu depends on the Zn content in our samples. As shown before, the Cu reduction occurs faster when no Zn is present at a current density of −500 mA^2^ cm^−1^. Furthermore, we can state that even after 1 h of CO_2_RR at a high current density, a certain amount of the Cu species present remains in the Cu(i) state, and the concentration of these species scales with the Zn loading. In the context of controlling the CO_2_RR selectivity, this observation is especially intriguing, when considering that the presence of cationic Cu species has been proposed to be the key for the excellent selectivity of oxide-derived copper catalysts toward ethanol and further reduced complex hydrocarbons.^13,25,30,44,51^ Moreover, we note here that the Cu reduction in our Zn/Cu_2_O NCs is noticeably slower than the reduction of Zn species. This observation is thus an interesting counterexample to the findings of our recent study for much smaller CuZn NPs, which was carried out at a much lower current density, where we have found that the Zn reduction lags behind the Cu reduction.^19^ This difference suggests that Cu and Zn reducibility trends are sensitive to the details of the selected electrochemical system and environment.

### Changes in Cu reduction kinetics: low current densities

Further insight into the effect of the amount of Zn in our system on the redox potentials of Cu is achieved by performing cyclic voltammetry. The presence of Zn has a major impact on the shape, intensity, and position of the Cu related CV features. We note here that the current densities achieved in the potential regions considered are much lower than those in our *operando* CO_2_RR measurements, namely between −3 and 3 mA cm^−2^. An important consequence is that a complete reduction or oxidation of Zn species is not achieved during these scans, thus the impact of the Zn species present on the Cu oxidation process is qualitatively different. In particular, we showed that in the case of the CV scans, the presence of coexisting Zn(0)/Zn(ii) species significantly increases the onset potential of the reduction of Cu(i) to metallic Cu as compared to the case of the pure Cu_2_O sample. Moreover, the presence of the Zn(0)/Zn(ii) species generally results in a noticeably lower concentration of Cu(i) species produced during the anodic part of the CV scan, as well as a delay in the formation of Cu(ii). The enhanced reducibility and hindered oxidation of Cu species in this case might stem from the strong oxidative character of Zn that donates electrons to Cu and enhances its reduction rate.

### Surface *vs.* bulk properties

From the results obtained during the CV scans, we conclude that the processes in the catalyst bulk and on the catalyst' surface may have drastically different kinetics and even different required redox potentials. In particular, from Zn K-edge QXAFS, we observe that the oxidation of Zn species during the anodic part of the CV scan takes place in two distinct steps. The first oxidation onset (feature A1, see Fig. 7) occurs in the potential range between −0.4 V and 0 V, and thus matches the expected oxidation potential for reduced Zn species (Zn(0) ↔ Zn(ii) + 2e^−^ = −0.3 V *vs.* RHE).^52^ We attribute this oxidation event to the oxidation of easily accessible near-surface Zn species. Meanwhile, the second oxidation peak for Zn species (feature A2 in Fig. 7) is at a significantly higher potential and does not match the aforementioned oxidation potential for Zn. As this second oxidation event happens concomitantly with the oxidation of Cu species, we hypothesize that due to (i) the oxidation of Cu species in an alloy structure, (ii) the dissolution of Cu(ii) under these conditions and (iii) resulting morphology changes, the Zn species, previously enclosed in a Cu-rich alloy, might become exposed to oxidative conditions, and get rapidly oxidized.

The difference in the oxidation state for surface and bulk species is also observed for copper. By comparing the results from QXAFS, SERS and cyclic voltammetry, we observed that the surface Cu species (probed by SERS) are reduced more easily (meaning at less negative potential) than bulk Cu species (probed by XAS). Moreover, while the reduction of bulk Cu species is enhanced by the presence of Zn species, the SERS features corresponding to the cationic Cu species on the catalyst surface disappear during the cathodic CV scan at practically the same potential for pure Cu_2_O and Zn/Cu_2_O catalysts. Overall, we conclude that the adsorbate structures on the catalyst surface and the kinetics of the surface processes are less affected by the presence of Zn species than the bulk structure and composition of our catalyst. Furthermore, in our SERS data, after exposing the catalyst to highly oxidative potentials during the CV scan, and thus, after partially oxidizing Cu to the Cu(ii) state, we observed the formation of a carbonate layer that remains stable also at negative potentials. In this regard, our study thus confirms the findings from ref. 44, where the prior oxidation of the catalyst to the Cu(ii) state resulted in the formation and stabilization of a carbonate passivation layer on the electrode surface under CO_2_RR conditions. According to our results, we can confirm that this layer is strictly limited to the catalyst surface as no carbonate species were detected in our bulk sensitive XAS data. Interestingly, we also see the formation of the carbonate layer before oxidizing our catalysts, which we attribute to the 10-fold higher KHCO_3_ concentration in our system compared to earlier studies.

We emphasize that only through the complementary application of time-resolved *operando* SERS and *operando* QXAFS measurements, coupled with advanced machine learning-based data analysis, a complete picture of the catalyst transformations under working conditions could be obtained. In particular, the machine learning approach allowed us for the first time to unambiguously distinguish between fcc (Cu-rich alloy) and non-fcc (Zn-rich alloy) type structures from EXAFS data in CuZnO_*x*_ materials and helped us to correlate the results of electrochemical characterization and electrocatalytic properties to the chemical and structural changes experienced by the catalytic system under working conditions. We found that the unique information from NN-EXAFS regarding the shifts in the RDF peaks (*i.e.*, changes in the average interatomic distances), is very informative in tracking the alloying/dealloying effects in these heterogeneous materials and complements well the conventional LCA-XANES analysis. Moreover, we highlight that this is the first time that the NN-EXAFS method was applied to the interpretation of QXAFS data. On one hand, due to the ability of the NN after the completed training to process the spectra very quickly (within seconds), this approach is well suited for deciphering large sets of spectra, which is typically the case for QXAFS measurements. On the other hand, we have demonstrated that the limited *k*-range and signal-to-noise ratio, characteristic for EXAFS spectra collected in an *operando* QXAFS mode, are not an obstacle for the NN-EXAFS approach. By validating the NN-EXAFS method on sets of well-defined experimental data, as well as by noting the excellent agreement between NN-EXAFS and LCA-XANES results, as observed in this study when deciphering *operando* QXAFS data, we have demonstrated the high robustness of the NN-EXAFS approach.

## 5 Conclusions

The results of this study provide us with crucial insights into the evolution of disordered Zn/Cu_2_O catalysts at high current densities and under potentiodynamic conditions. In particular, through the combination of *operando* QXAFS and SERS methods and advanced data analysis, we were able to track the evolution of the complex heterogeneous mixture of different species comprising our bimetallic catalytic system. Moreover, we decoupled the contributions of oxidized and reduced species, alloyed and segregated phases, those of species with fcc- and non-fcc-type structures, as well as of surface and bulk species. The extracted structural and chemical information can be directly correlated to the catalytic performance of our materials. We conclude that the presence of the Zn-rich hcp-type structures prompts increased hydrogen and CO formation, while dilute Cu-rich fcc-type structures, enriched by cationic sub-surface Cu(i) species, are favorable for C–C coupling and, hence, for the CO_2_ conversion to valuable multicarbon products.

## Author contributions

M. R., J. T. and B. R. C. co-wrote the paper. A. H. and H. S. J. prepared the samples and A. H. performed the *ex situ* characterization and *operando* SERS measurements, while H. S. J. performed the electrochemical measurements. M. R. and J. T. designed the synchrotron experiments. M. R., A. H., H. S. J. and J. T. conducted the synchrotron experiments. M. R. and J. T. analyzed XAS data. J. T. designed and employed the NN-EXAFS method. W. F. and S. K. performed the TEM measurements. B. R. C. supervised the study. All authors participated in the discussion of the obtained results and editing of the manuscript.

## Conflicts of interest

There are no conflicts to declare.

## Supplementary Material

CY-012-D2CY00227B-s001
