# Supplementary material for: Tracking heterogeneous structural motifs and the redox behaviour of copper–zinc nanocatalysts for the electrocatalytic CO2 reduction using operando time resolved spectroscopy and machine learning
Source: Catal Sci Technol. 2022 Mar 10;12(9):3028–43. doi: 10.1039/d2cy00227b (PMC9089751; doi:10.1039/d2cy00227b)
Supplement: CY-012-D2CY00227B-s001 [file CY-012-D2CY00227B-s001.pdf]

Electronic Supplementary Material (ESI) for Catalysis Science & Technology.
This journal is © The Royal Society of Chemistry 2022

Supplementary Information

Tracking heterogeneous structural motifs and the redox behaviour of copper-zinc nanocatalysts for the electrocatalytic CO_2 reduction using operando time resolved spectroscopy and machine learning

Martina Rüscher , Antonia Herzog , Janis Timoshenko* , Hyo Sang Jeon, Wiebke Frandsen, Stefanie Kühl and Beatriz Roldan Cuenya *

Department of Interface Science, Fritz-Haber Institute of the Max-Planck Society, 14195 Berlin, Germany

*Corresponding authors. E-mails: roldan@fhi-berlin.mpg.de, janis@fhi-berlin.mpg.de

Supplementary Note 1: synthesis of the Zn/Cu_2O nanocubes

ZnO decorated Cu_2O nanocubes (NCs) were prepared by using a wet-chemical ligand-free method, which is similar to synthesis routes reported before.[1, 2] All reagents were purchased from Sigma-Aldrich in ACS grade and used without further purification. 5 mL of an aqueous $CuSO_4$ solution (0.1 M) were mixed at room temperature with 457.5 mL of ultrapure water (> 18 MΩ cm^{-1}). Then, 17.5 mL of an NaOH solution (1.0 M) were injected under continuous stirring. After 5 s, 20 mL of an L-ascorbic acid solution (0.25 M) were added to the reaction mixture and stirred for 12.5 min. Afterwards, a mixture of 3.3/5/20 mL of L-ascorbic acid (0.25 M) and of 1.7, 2.5 or 10 mL of $ZnCl_2$ solution (0.1 M) was added to the reaction solution to obtain the catalysts with 4, 7 or 15 % Zn loading, respectively. The solution was then stirred for one additional minute. The solution was centrifuged and washed three times, twice with an ethanol-water mixture (1:1) and twice with pure ethanol. The final product was dried in vacuum overnight. The bare Cu_2O NCs as reference were prepared in the same way without adding the mixture of L-ascorbic acid and $ZnCl_2$.

The working electrodes were prepared by mixing 12.5 mg of the catalyst powder with 2 mL of methanol and 80 µL of a Nafion solution (Sigma-Aldrich, ~ 5 wt% in a mixture of alcohols and water). The mixture was ultrasonicated for at least 30 min, and 2 mL of the catalyst dispersion were spray-coated on the microporous layer of a 5 cm x 5 cm carbon-based gas diffusion electrode (GDE, Sigracet 28BC), which was placed on a hot plate at 120°C.

Supplementary Note 2: surface enhanced Raman spectroscopy measurements

For the SERS measurements, a Si(100) wafer (520.5 cm^{-1}) served as calibration substrate. Further, a near-infrared laser (Renishaw, RL785, λ = 785 nm, P_{max} = 500 mW) was used as excitation source. The backscattered light was Rayleigh-filtered, and the Raman scattering was collected in the range of 100–1200 cm^{-1} with a grating of 1200 lines mm^{-1} and guided to a CCD detector (Renishaw, Centrus). For the *operando* measurements, the excitation source was focused on the surface of the sample and Raman scattering signals were collected with a water immersion objective (Leica microsystems, 63×, numerical aperture 0.9) protected from the electrolyte by a Teflon film (DuPont, film thickness of 0.013 mm) wrapped around the objective. The acquisition of each spectrum was performed with 5 s of exposure time. The Raman data were processed using the Renishaw WiRE 5.2 software. The spectra were baseline-subtracted using the intelligent spline feature of eighth order, and cosmic rays were removed.

Supplementary Note 3: electrochemical characterization

The following equation (Eq. S1) was used to convert the measured potentials (V) to the reversible hydrogen (RHE) electrode scale:

$$E(V\ vs.\ RHE) = E\ (V_{measured} vs. Ag/AgCl) + 0.242 + 0.059 * pH_{bulk\ electrolyte}$$

The pH of the bulk electrolyte was determined to be 7.8.

Supplementary Note 4: XAS measurements and data analysis

The QXAFS (Quick X-ray absorption fine structure) measurements were conducted at the SuperXAS – X10DA beamline at the SLS synchrotron facility of the Paul Scherrer Institute. For the energy selection

a channel-cut LN-cooled Si(111) monochromator was used, which was oscillating with 1 Hz frequency. Rh-coated collimating mirrors and Rh-coated focusing mirrors were used to reject higher harmonics and reduce the heat load on the sample. The intensity of the incident radiation was measured using ionization chambers filled with N_2. *Operando* XAS measurements were performed in fluorescence mode using a PIPS detector. The measurements were performed at the Cu K-edge (8979 eV) and the Zn K-edge (9659 eV).

For *operando* XAS measurements, we used our home-built gas-fed cell, which is suitable for measurements at high current densities. The cell design is described in Ref. [3] Samples were spray coated on a GDE with an area of 1 cm^2. The sample loading was optimized and the sample amount for spray coating on the GDE was increased by a factor of 2.4, compared to the sample amount used for electrocatalytic measurements, to ensure a sufficient signal to noise ratio at the Cu K-edge and the Zn K-edge, but simultaneously avoid self-absorption effects.

For the calibration, a Cu foil spectrum was collected at the beginning of each QXAFS scan before moving the cell into the X-ray beam. The data calibration was performed with the Pro-QEXAFS software [4]. Further data processing, data reduction, spectra normalization, averaging and linear combination analysis of the XANES spectra were performed using a set of home-built Wolfram Mathematica scripts. The EXAFS data were extracted from averaged XAS spectra using the Athena software.[5] Advanced EXAFS data processing was carried out using a neural network approach as discussed in Supplementary Note 5.

Supplementary Note 5: Machine learning – description, validation, and analysis at different current densities

To develop the neural network for the analysis of our EXAFS spectra for mixtures of Zn species, we followed a similar procedure as discussed in our previous works.[6, 7] Here, the important difference is that instead of extracting the partial radial distribution functions (RDFs) from the EXAFS data for atoms of different types, we extracted the partial radial distributions corresponding to the different Zn-containing phases (see the discussion in the main text). Briefly, we designed a neural network (NN), a composite mathematical function $h(\chi(k),\vec{\theta})\rightarrow w_{oxide}g_{oxide}(R), w_{fcc}g_{fcc}(R), w_{non-fcc}g_{non-fcc}(R)$, that maps an EXAFS spectrum $\chi(k)$ to the set of RDFs $g_{oxide}(R)$, $g_{fcc}(R)$, and $g_{non-fcc}(R)$ corresponding to oxide phase, metallic fcc-like phase and metallic non-fcc-like phase of Zn, respectively. w_{oxide}, w_{fcc} and $w_{non-fcc}$ are the relative weights of the corresponding phases. As in our previous works, the EXAFS spectrum $\chi(k)$ was discretized by performing the Morlet wavelet transformation,[6, 8] and providing selected wavelet coefficients as an input for the NN. For the analysis in this work, we used a relatively short spectral range in *k*-space (between 3 and 8 $Å^{-1}$). The total input vector for NN contained 738 points. The RDFs were approximated with a histogram of interatomic distances, calculated in the range between R_{min} = 1.0 Å and R_{max} = 5.5 Å, with the histogram bin width 0.04 Å. The NN output vector thus contains 336 points. In-between input and output layers, our NN contained three hidden layers with 1000 nodes each to ensure sufficient flexibility of our model.

The parameters of the NN nodes (weights and biases) were found during the training step, where we provided the theoretical spectra with a known structure as an input for the NN and tuned the parameters to ensure a good agreement between the NN outputs and the true RDFs for our models. For the training, we used linear combinations of EXAFS spectra, which were obtained from molecular dynamics (MD) and Monte Carlo (MC) simulations using empirical force field models. These simulations

have been carried out as described in our previous work.[6] As we have demonstrated in Figure S14a, the obtained theoretical spectra resemble well the experimental data of reference compounds, giving us confidence that after the training using theoretical data, the NN will be able to recognize features in our experimental data of the investigated samples as well. Note that the RDFs (Figure S14b), obtained in these simulations for ZnO, metallic Zn and CuZn brass alloy, are clearly distinct. For instance, while the contribution of the first coordination shell appears to be similar for metallic Zn and CuZn brass structures (featuring distorted hcp-type structure and fcc-type structure, respectively, both having 12 nearest neighbours around the central atom, see Figure S14c), the peaks in the RDF region between 4 and 6 Å are very different. This suggests that there is sufficient contrast between these structures so that their amounts in the mixture can be quantified reliably by our EXAFS analysis, if it is extended to the contributions of these distant coordination shells. We would like to stress here, that this is challenging to do in conventional EXAFS fitting, but it is straightforward in NN-EXAFS approach.

For the NN training, the numbers of the theoretical spectra that we used are the following: 1512 spectra calculated for metallic Cu, 1804 spectra for Cu_2O, 1708 spectra for CuO, 1231 spectra for $Cu(OH)_2$, 1512 spectra for hcp-type Zn, 1320 spectra for wurtzite-type ZnO, 772 spectra for rocksalt-type ZnO. Different spectra were obtained by changing the temperature and lattice spacings in the MD/MC models to extend the applicability of our approach to a broad range of metals, oxides, and alloys with different degree of disorder and different lattice constants. These unique spectra were further combined linearly to form 60000 model spectra for mixtures, which then were used for the NN training. We explained the technical details of the NN implementation and training in our previous work[5]. To estimate the uncertainties, we created 10 NNs, which were trained independently on different training sets. The results that are reported in this study are an average result, given by these 10 NNs, and the standard deviation of their predictions is used as an estimate of the random uncertainty of the NN-EXAFS method.

After the training was completed, the NN parameters were fixed, and the NN could be used to process experimental EXAFS spectra. Before applying the NN to the interpretation of our *operando* data, we first tested the NN on experimental data for well-defined reference compounds, for which the particular RDFs can be obtained independently from the EXAFS data using the reverse Monte Carlo (RMC) approach.[9-11] As shown in Figure S15ab, the RDFs, which were obtained by the NN-EXAFS method, are in an excellent agreement with the RMC results in the cases where we applied the method to EXAFS data for fcc metals with different lattice constants (metallic Cu, Ni, CuZn brass), EXAFS data for metals with distorted hcp-structure (metallic Zn), as well as to EXAFS spectra in different oxides (Cu_2O, CuO, ZnO). Furthermore, to test the applicability of our method for the speciation of mixtures, we constructed artificial linear combinations of experimental Zn K-edge EXAFS spectra for Zn metal, wurtzite-type ZnO, and CuZn, where the spectra were added with random weights. We then provided these spectra as an input to our NN and estimated the mixing weights of these three components by using Eq. (2) in the main text. As one can see from Figure S15cde, the NN results are in a good agreement with the known true values of the weights for oxide, fcc- and hcp-phases in the mixtures. We estimated that the average deviation between the true value of the species' concentration and the NN result is ca. 4.4% for the oxide phase, 7.7% for the fcc phase, and 7.6% for the hcp phase. These numbers represent the expected systematic uncertainty of our NN-EXAFS method for the speciation of Zn phases.

Examples of RDFs that were reconstructed for these mixtures of reference spectra, are shown in Figure S16. The obtained RDFs are compared here with the linear combination of RDFs, obtained for

the reference structures by RMC method, where the combined RDFs are weighted with the same weights as reference spectra in the analyzed mixture spectrum. From this figure, one can confirm that not only the weights of different phases are reproduced with reasonable accuracy, but also the RDFs themselves. This indicates that we can reliably extract information on the distribution of interatomic distances from our NN-EXAFS method. Therefore, our validation procedure thoroughly demonstrates that the changes in interatomic distances due to alloying/dealloying processes can be tracked accurately by our approach.

After the described training and validation of the NN with experimental data for reference compounds, this method then can be applied to the interpretation of *operando* QXAFS spectra for our catalysts.

Supplementary Note 6: Interpretation of CV features corresponding to catalyst oxidation

In the performed cyclic voltammetry (CV) scans, we observed several features that can be attributed to the oxidation of the catalyst species (see Figure 7 in the main text). The main oxidation features for Cu_2O catalyst are marked as B1 and B2 and correspond to the CV peaks visible at ca. 0.7 V in the anodic scan (B1), and at ca. 0.5 V (B2). The main features in the anodic part of CVs for the bimetallic catalysts are marked as A1 and A2. A1 corresponds to the region between -0.4 V and 0 V in the anodic scan, where two small split peaks are observed in the CVs for samples with 4% and 7% Zn loading, and one broad peak for the 15% Zn containing sample. A2 denotes the intense peak followed by a current plateau from 0.5 V until the upper vertex in the upward scan. To identify these features, we used XAS and LCA-XANES and NN-EXAFS analyses.

Feature A1 in the CVs for bimetallic samples appears simultaneously with the contribution of Zn(II) in LCA-XANES data as well as with the appearance of the oxide structure contribution in the NN-EXAFS data of the Zn K-edge. This feature can thus be linked to the oxidation of Zn species in a CuZn alloy and a metallic phase. Indeed, this feature is not present in the CV for pure Cu_2O NCs, and its intensity increases systematically with the Zn loading (Figure S20). Moreover, the appearance of this feature also coincides with the onset of the decrease of the Zn fluorescence intensity (Figure S21). We attribute the decrease of the Zn fluorescence signal to the dissolution of newly formed cationic Zn species, which are expected to be unstable at these potentials.[12] Both, the metallic Zn phase and the CuZn alloy phases with different Cu to Zn ratios are affected by the oxidation of the sample. Thus, Zn species with different local structure and charge state will be present, which likely explains why the A1 peak is broad and split in several small peaks for samples with lower Zn loading. The NN-EXAFS analysis also shows that the oxidation of Zn is accompanied by a shift of the RDF peaks in both, fcc and non-fcc phases, to lower interatomic distances (Figure S25), suggesting that the oxidation is accompanied by dealloying processes, forming increasingly copper-rich phases. Note that at these potentials the Cu species remain metallic.

Features B2 and A2 are among the most pronounced features in the CVs for pure Cu_2O, and for the Zn-decorated samples, respectively. The onset potentials of B2 and A2 peaks are similar, but the A2 peaks are much broader for Zn-decorated samples. In both cases, we attribute this feature to the oxidation of metallic Cu to the Cu(I) state. We note, nonetheless, that the oxidation of Cu to the Cu(II) state also contributes to this feature. Indeed, our LCA-XANES

results show that for the monometallic Cu sample, the signatures of Cu(II) species appear almost simultaneously with those of Cu(I) species, suggesting that oxidation of Cu(0) species to Cu(I) and Cu(II) takes place simultaneously. At the same potential, we also observe the onset of the decrease in the Cu fluorescence signal (Figure S27), which based on our previous works can be assigned to the dissolution of newly formed, unstable Cu(II) species.[3, 12] We note that the decrease of Cu fluorescence continues during the CV scan, until the potential is lowered and Cu(II) species are reduced. The remaining Cu(I) and Cu(0) species, unlike Cu(II) species, are stable and are not dissolving.

One can note that the oxidation of Cu proceeds clearly differently in the bimetallic samples than in the monometallic sample. First of all, the overall amount of Cu(I) species formed during the CV scan at potentials above 0.5 V is much lower in the bimetallic catalysts than for the pure Cu_2O NCs. An increased Zn content, however, increases the amount of Cu(I) species formed in the bimetallic samples. This can be related to the higher contribution of residual Cu(I) species with increasing Zn loading at more negative potentials before and after the oxidation cycle (for pure Cu_2O NCs: 4% Cu(I), while for the sample containing 15% of Zn: 12% Cu(I)). Moreover, the appearance of the Cu(II) contribution is slightly delayed in the bimetallic samples. We attribute both these effects to the competition between the oxidation of Zn species and Cu species. Indeed, in bimetallic catalysts, the Cu oxidation takes place on the background of continuous Zn oxidation. Moreover, the feature A2 further marks the onset of a significant boost in the oxidation of Zn species, as visible from our LCA-XANES and NN-EXAFS results. We explain this sharp increase in the Zn oxidation by the fact that the oxidation of Cu exposes the Zn species to oxidative conditions, which were so far protected from the oxidation by being incorporated in the bulk of CuZn alloy phases. Simultaneously, the oxidation of these Zn species could delay the oxidation of Cu to the Cu(II) state. Interestingly, while the appearance of Cu(II) species is shifted to a higher potential in the bimetallic catalysts, the amount of Cu(II) generated is larger than in the monometallic catalyst and systematically increases with Zn loading. This effect can be tentatively attributed to the fact that the lower contribution of Cu(I) species observed in the bimetallic catalysts leaves more metallic Cu sites to be directly oxidized to the Cu(II) state. The combination of these three aforementioned effects in our bimetallic catalysts: (i) a more pronounced Cu oxidation to Cu(II) state at higher Zn loadings, (ii) the shift of the onset of Cu oxidation to Cu(II) species to higher potential with respect to that in the monometallic catalysts, and (iii) the additional contribution of Zn species oxidation explains why the A2 feature in our bimetallic catalysts is much broader than the corresponding B2 feature in the monometallic Cu_2O catalyst.

For the discussion of CV features observed during the cathodic part of CV scan the discussion can be found in the main text.

Supplementary Figures

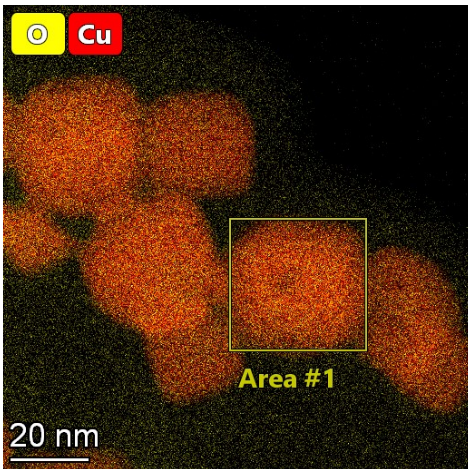

Figure S1. STEM-EDX image of a Cu_2O NC sample in as-prepared state with the area indicated that was used for the quantification of the Cu:O ratio.

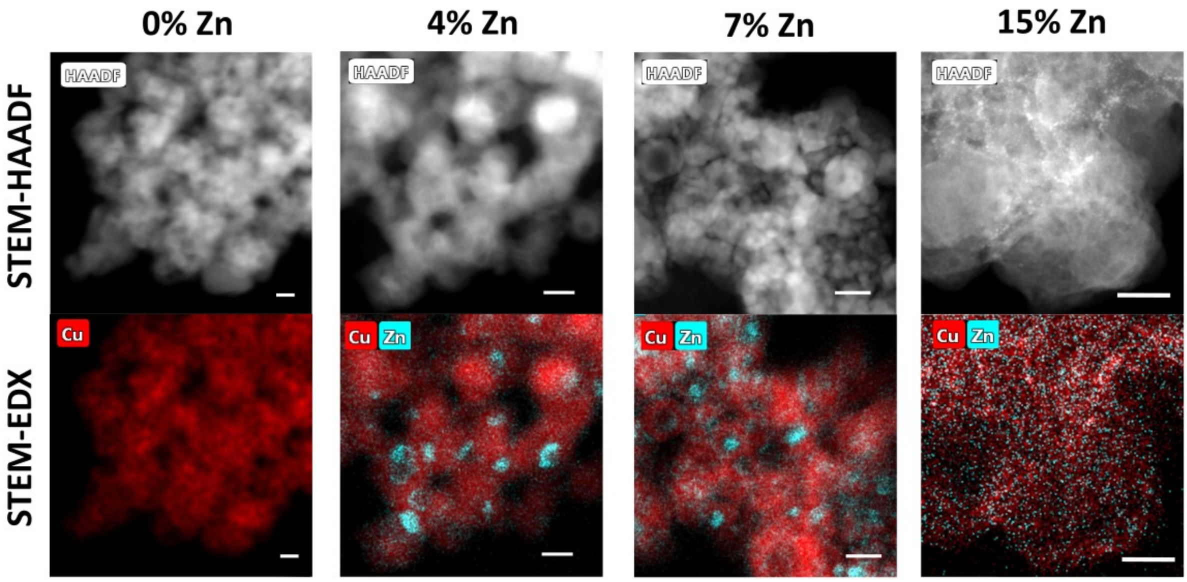

Figure S2. STEM-EDX and STEM-HAADF images of the Cu_2O NC sample and of the bimetallic samples with different Zn loadings (4 %, 7 % and 15%) after 50 minutes of CO_2RR at a current density of -500 mA/cm². The white bars indicate the scale bar length of 20 nm.

Table S1. Edge lengths and the corresponding size distribution of Cu_2O NCs obtained from the analysis of STEM-HAADF images and the dimension of the Zn shells/islands obtained from the analysis of the corresponding EDX maps of the bimetallic samples containing 4%, 7% and 15% Zn. Data for each sample are included in their as-prepared state as well as after CO_2RR at -500 mA/cm². For the CuZn sample containing 15% Zn, no formation of Zn islands was observed after the exposure to CO_2RR conditions.

sample	as-prepared		after CO_2RR	
	Cu_2O NCs (nm)	Zn shell (nm)	Cu_2O NCs (nm)	Zn island (nm)
**0% Zn**	23.3 ± 5.3	-	31.8 ± 9.1	-
**4% Zn**	21.6 ± 4.3	> 1	28.3 ± 5.8	7.1 ± 2.7
**7% Zn**	22.2 ± 3.8	≥ 2	26.3 ± 5.9	8.1 ± 3.0
**15% Zn**	25.3 ± 4.5	≥ 2	30.4 ± 7.2	-

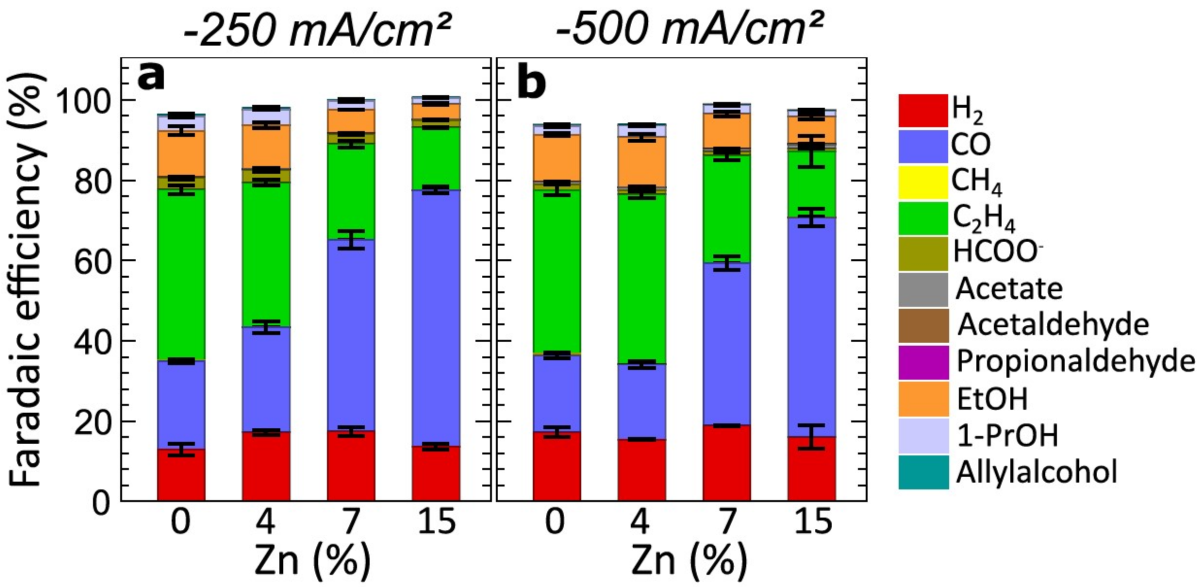

Figure S3. Faradaic efficiencies (FEs) for our Zn/Cu_2O samples with different Zn loadings after 50 minutes of CO_2RR at a current density of (a) -250 mA/cm² and (b) -500 mA/cm².

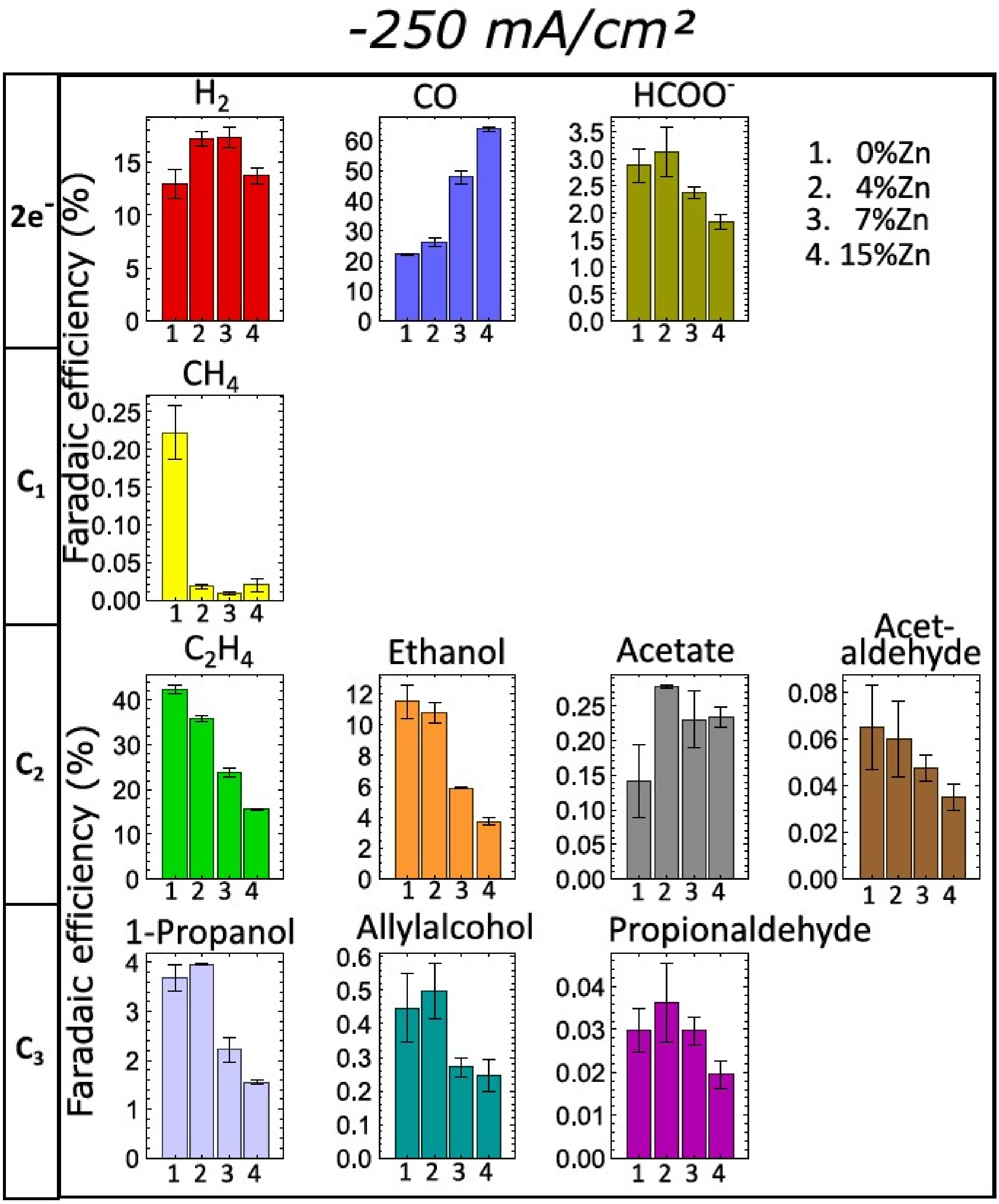

Figure S4. Comparison of the FEs of the main reaction products for the Cu_2O NCs and the bimetallic Zn/Cu_2O NCs samples with different Zn loadings obtained for CO_2RR in 1 M $KHCO_3$ at a current density of -250 mA/cm². Results for 2e- transfer products (hydrogen, carbon monoxide and formate), further reduced C_1 products (methane), C_2 products (ethylene, ethanol, acetate, acetaldehyde) and C_3 products (1-propanol, allyl alcohol, propionaldehyde) are shown separately.

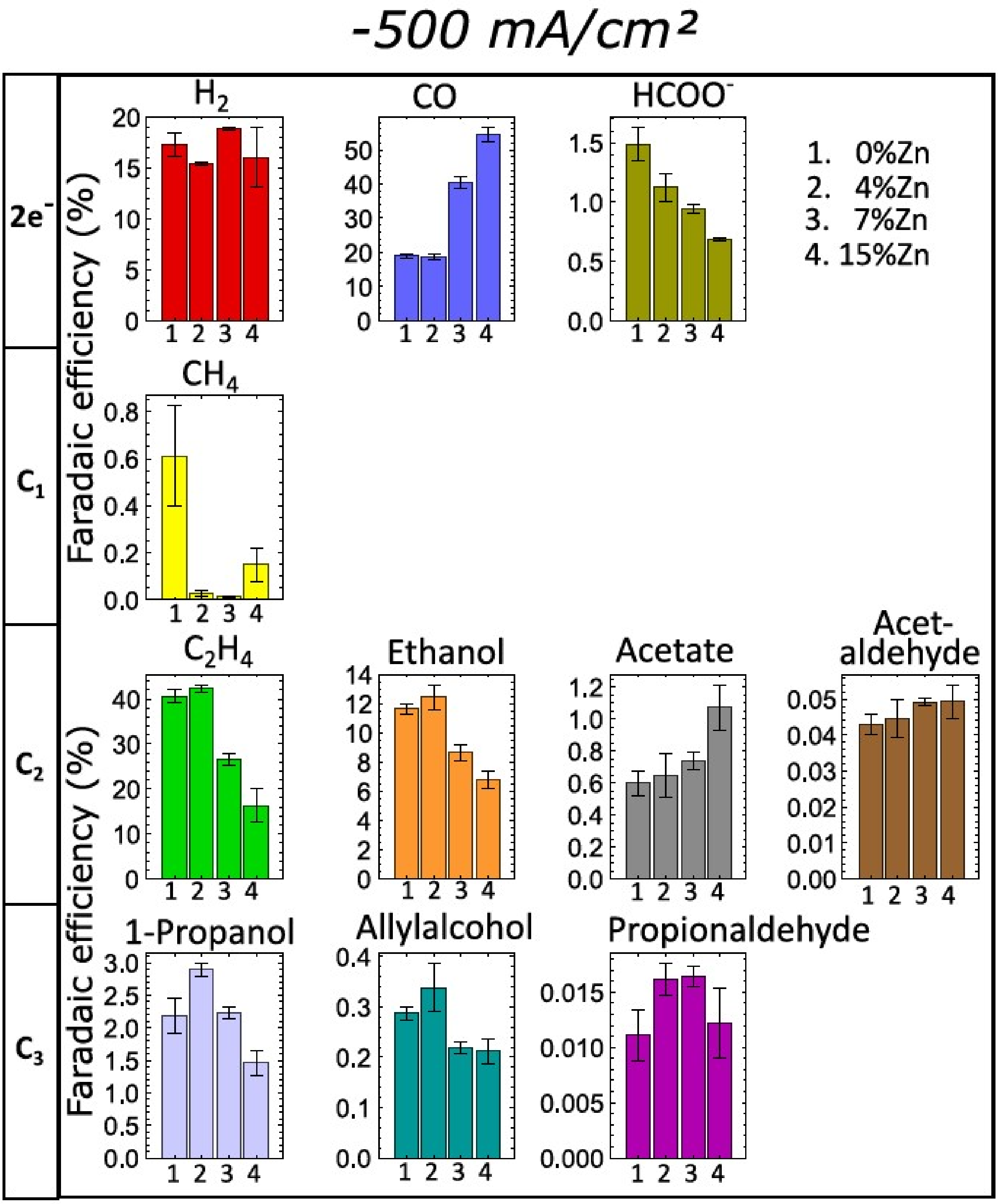

Figure S5. Comparison of the FEs of the main reaction products for the Cu_2O NCs and the bimetallic Zn/Cu_2O NC samples with different Zn loadings obtained for CO_2RR in 1 M $KHCO_3$ at a current density of -500 mA/cm². Results for 2e- transfer products (hydrogen, carbon monoxide and formate), further reduced C_1 products (methane), C_2 products (ethylene, ethanol, acetate, acetaldehyde) and C_3 products (1-propanol, allyl alcohol, propionaldehyde) are shown separately.

Table S2. Reaction rates of the main CO_2RR products and total CO_2RR reaction rates at -250 mA/cm² and -500 mA/cm².

current density		-250 mA/cm²				-500 mA/cm²			
sample		0% Zn	4% Zn	7% Zn	15% Zn	0% Zn	4% Zn	7% Zn	15% Zn
reaction rates (10^{-8}*mol*cm^{-2}*s^{-1})	CO	7.9(1)	9.5(5)	17.1(8)	22.9(3)	13.7(5)	13.4(7)	29(1)	39(2)
	C_2H_4	2.54(6)	2.16(4)	1.43(5)	0.93(1)	4.8(2)	5.1(1)	3.2(1)	2.0(5)
	$HCOO^-$	1.0(1)	1.1(2)	0.85(4)	0.66(5)	1.1(1)	0.81(8)	0.68(3)	0.49(1)
	Ethanol	0.69(6)	0.65(4)	0.35(0)	0.22(2)	1.40(4)	1.5(1)	1.04(6)	0.82(7)
	1-Propanol	0.15(1)	0.16(0)	0.09(1)	0.06(0)	0.17(2)	0.23(1)	0.18(1)	0.17(2)
	all CO_2RR products	12.4(4)	13.6(8)	20.0(9)	24.9(4)	22(1)	21(1)	34(1)	42(2)
	all liquid products	0.87(9)	0.86(5)	0.48(2)	0.32(2)	1.71(8)	1.9(1)	1.38(8)	1.6(1)

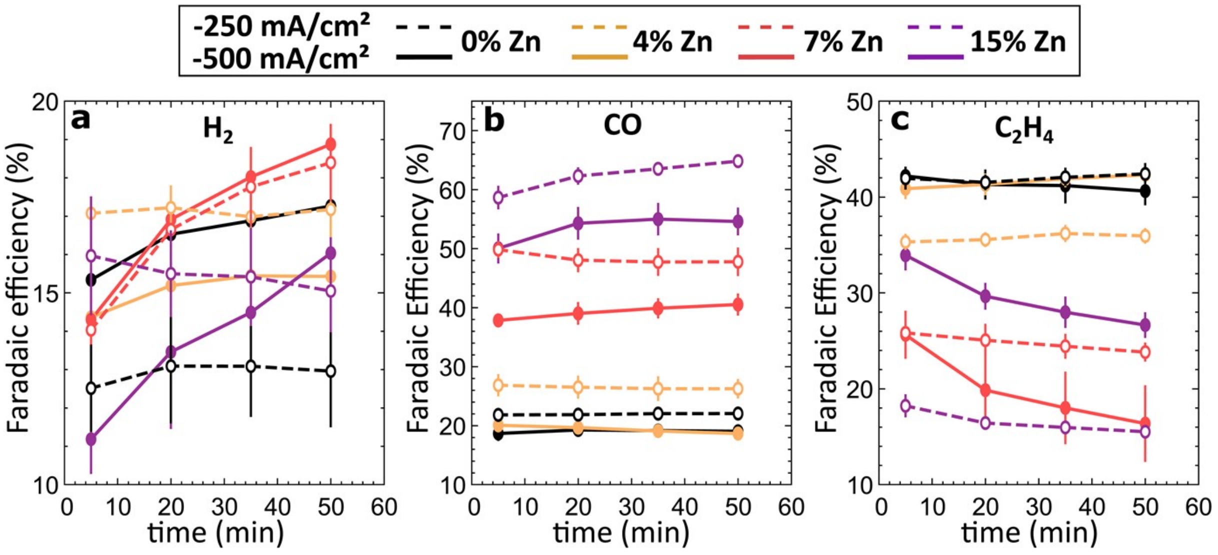

Figure S6. The evolution of the FEs for the main gaseous products: hydrogen (a), carbon monoxide (b) and ethylene (c) during 50 minutes of CO_2RR at a current density of -250 mA/cm² (dashed lines) and of -500 mA/cm² (solid lines). Products were detected every 15 minutes with a gas chromatograph.

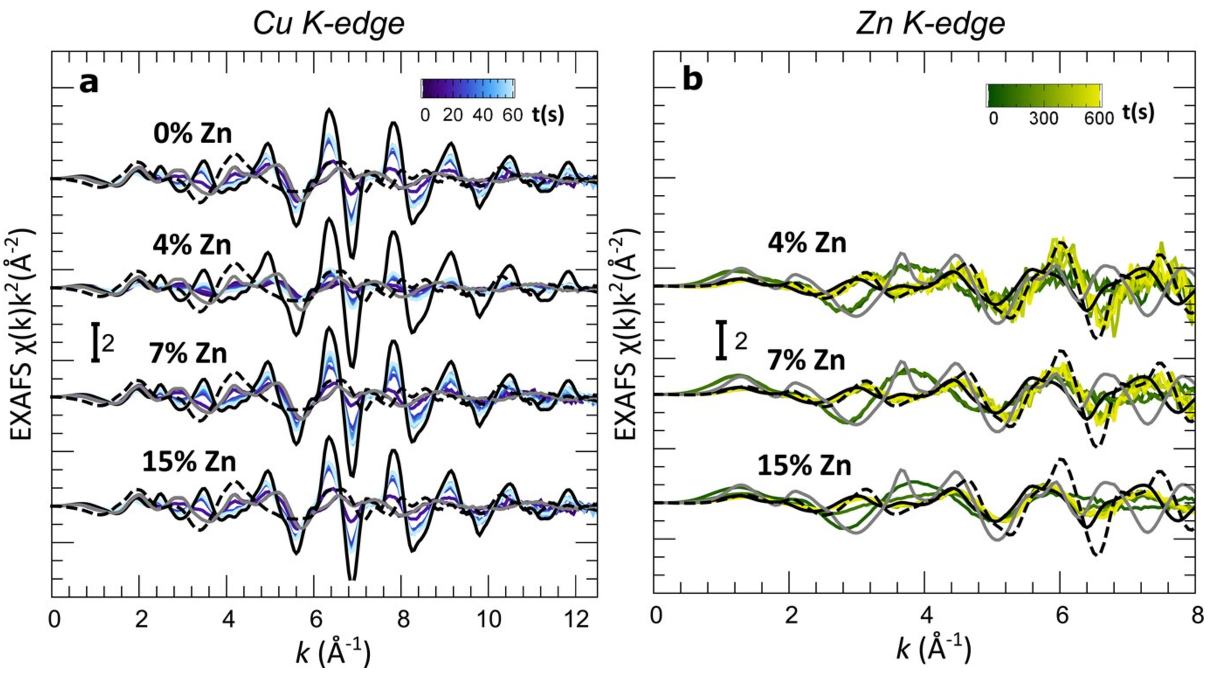

Figure S7. EXAFS data for the pure Cu_2O NCs and the bimetallic Zn/Cu_2O NCs samples with different Zn loadings, collected under CO_2RR at a current density of -500 mA/cm^2. Evolution of the EXAFS spectra at the Cu K-edge (a) and the Zn K-edge (b) is shown. The depicted Cu K-edge spectra were extracted from our QXAFS data that were averaged for 10 s, while the Zn K-edge spectra were extracted from our QXAFS data averaged for 100 s due to the significantly lower loading of Zn as compared to Cu. The spectra are shifted vertically for clarity. The reference Cu K-edge spectra for bulk Cu_2O (dashed black line in (a)) and Cu foil (solid black line in (a)), as well as the Zn K-edge reference spectra for ZnO (solid gray line in (b)), Zn foil (solid black line in (b)) and CuZn brass foil (dashed black line in (b)) are shown for comparison.

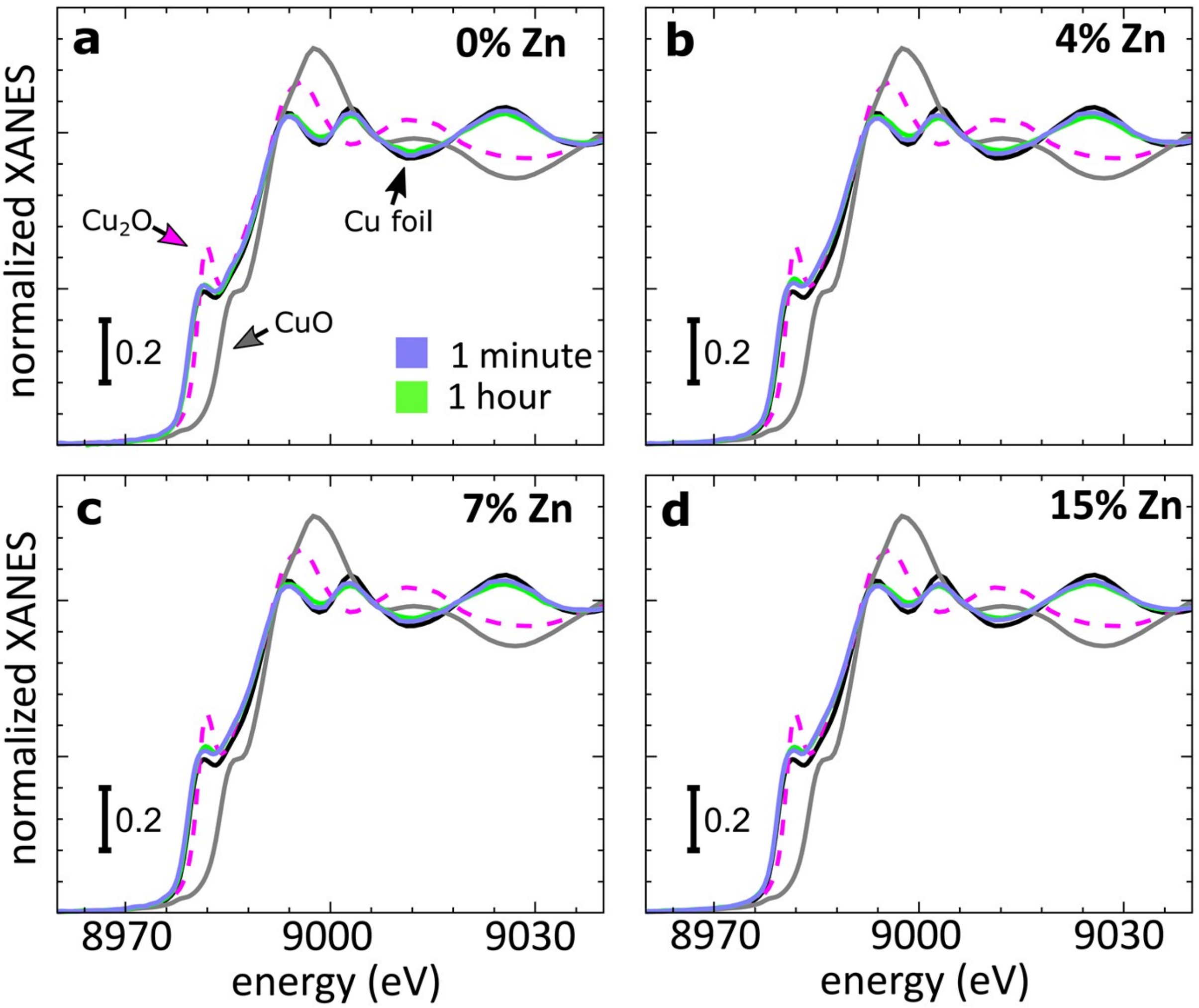

Figure S8. Cu K-edge XANES spectra collected after 1 minute and 1 hour of CO_2RR at a current density of -500 mA/cm² for a pure Cu_2O NC catalyst (a) and for a Zn/Cu_2O NC catalysts with the Zn loadings 4% (b), 7% (c) and 15% (d). The reference Cu K-edge spectra for CuO (solid gray line), Cu foil (solid black line) and bulk Cu_2O brass foil (dashed magenta line) are shown for comparison.

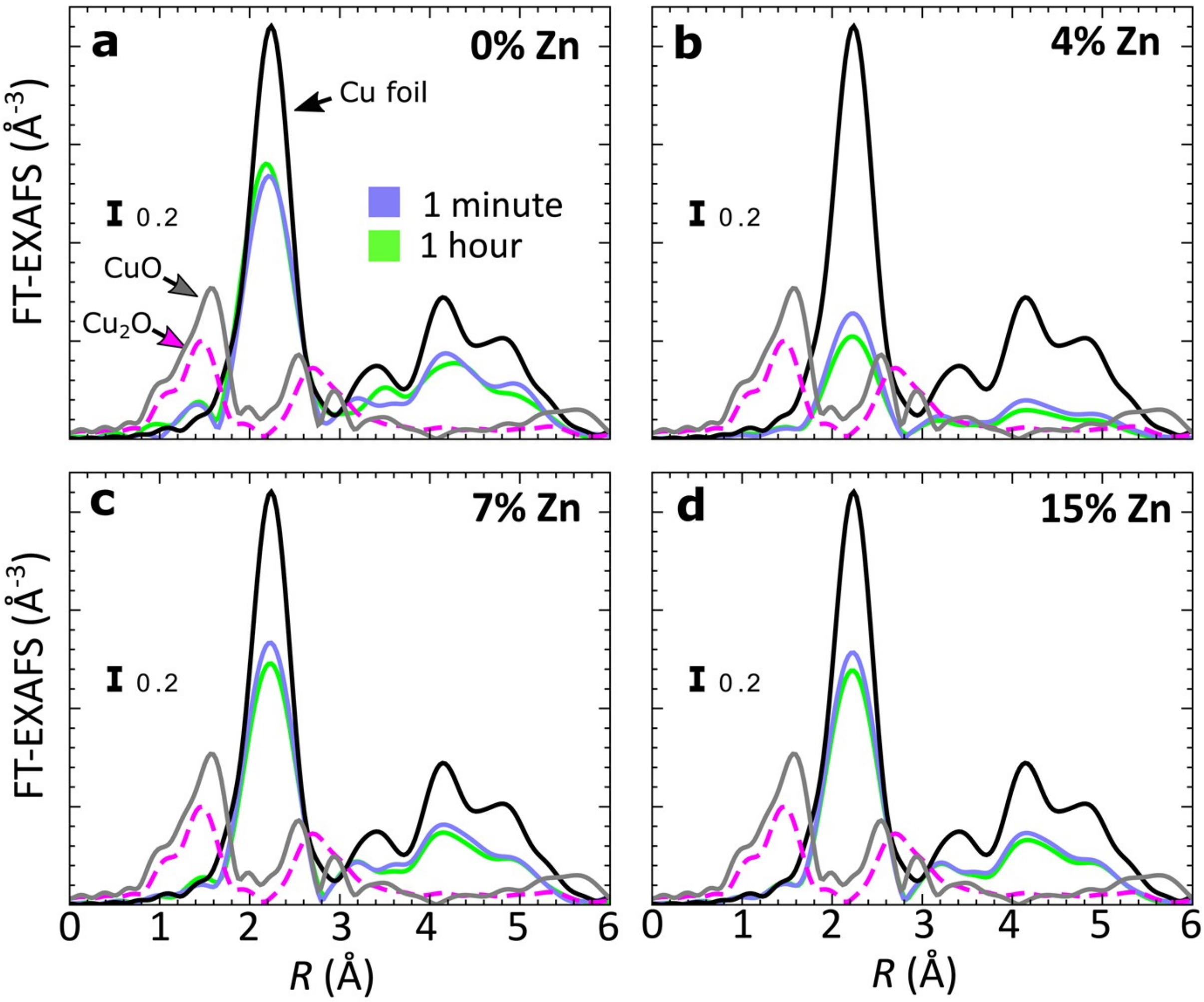

Figure S9. Cu K-edge Fourier-transformed (FT) EXAFS spectra collected after 1 minute and 1 hour of CO_2RR at a current density of -500 mA/cm² for pure Cu_2O NC catalyst (a) and for Zn/Cu_2O NC catalysts with the Zn loadings 4% (b), 7% (c) and 15% (d). The reference Cu K-edge spectra for CuO (solid gray line), Cu foil (solid black line) and bulk Cu_2O brass foil (dashed magenta line) are shown for comparison.

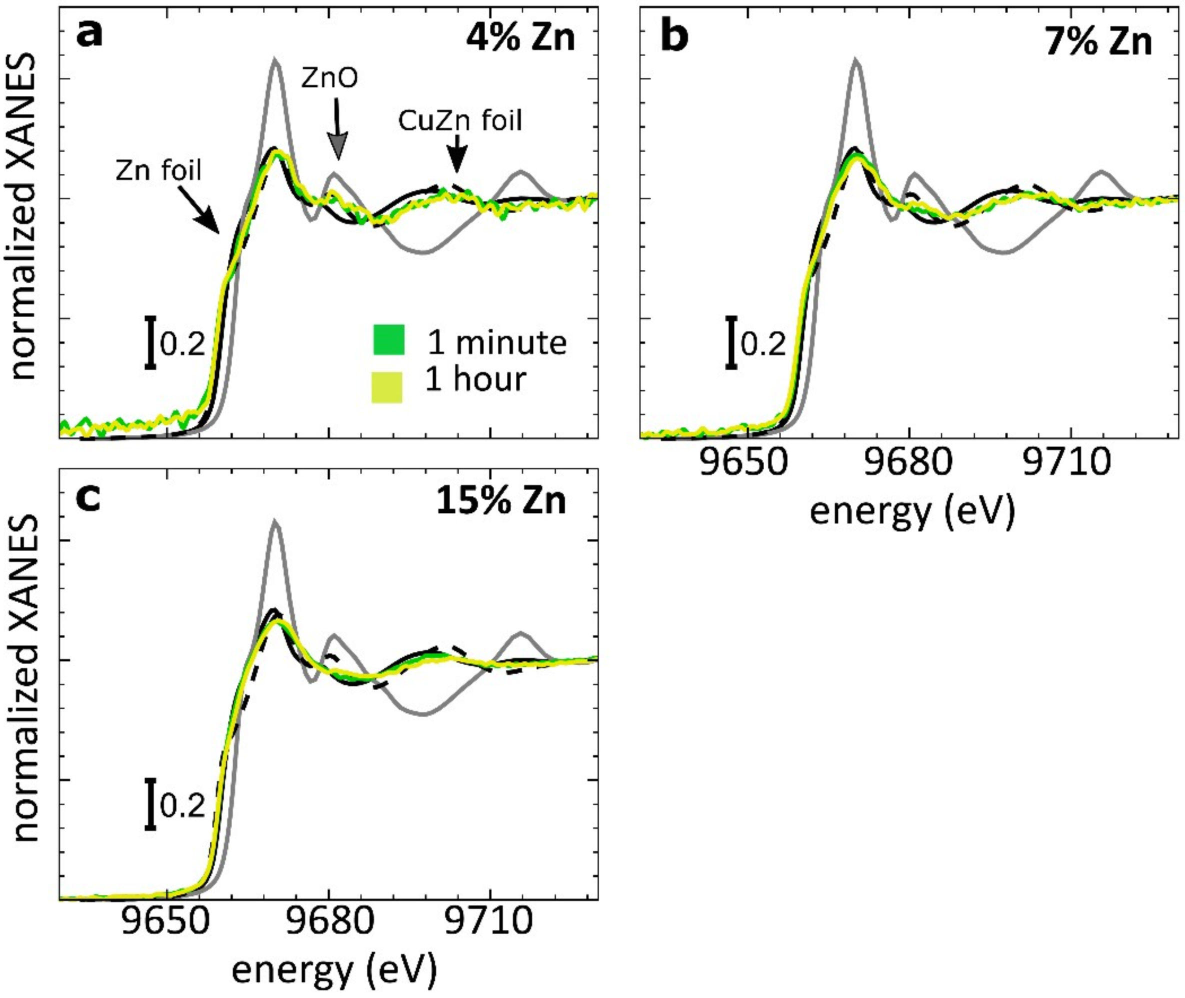

Figure S10. Zn K-edge XANES spectra collected after 1 minute and 1 hour of CO_2RR at a current density of -500 mA/cm² for Zn/Cu_2O NC catalysts with Zn loadings 4% (a), 7% (b) and 15% (c). The reference Zn K-edge spectra for bulk ZnO (solid gray line), Zn foil (solid black line) and CuZn brass foil (dashed black line) are shown for comparison.

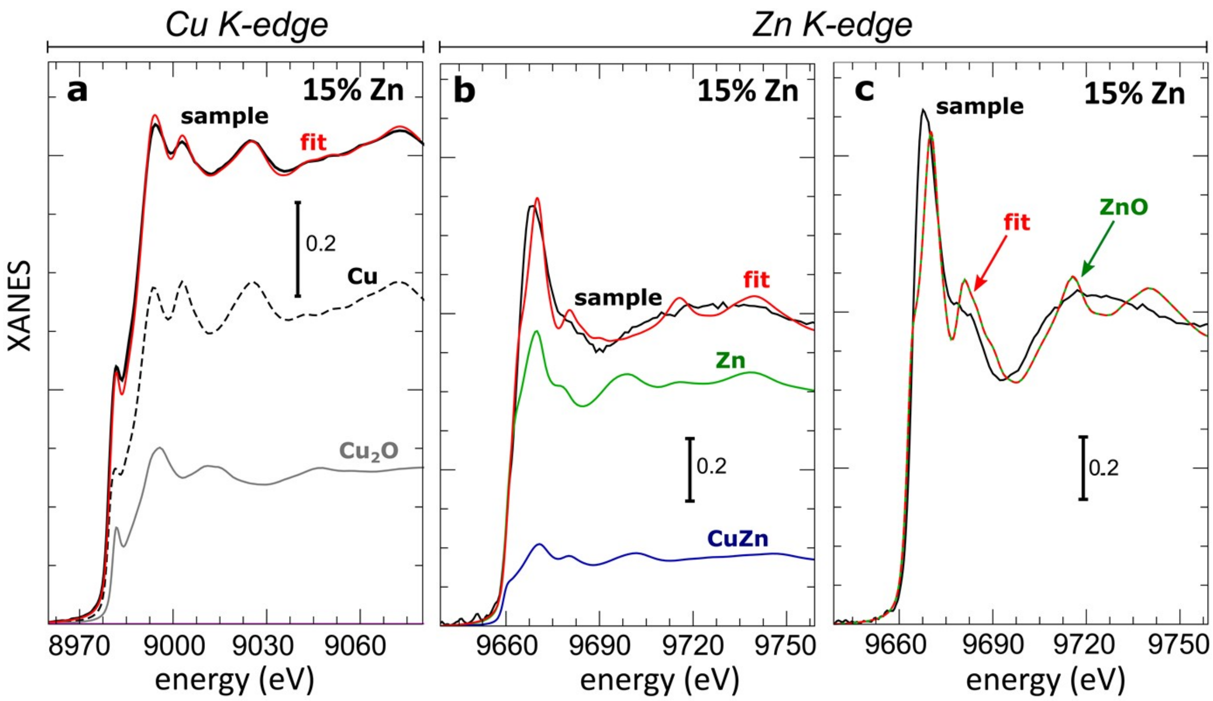

Figure S11. Exemplary LCA fits of the XANES spectra at (a) the Cu K-edge and (b) the Zn K-edge of the Zn/Cu_2O NCs sample containing 15% of Zn measured 10 s after applying a current density of -500 mA/cm² under CO_2RR conditions.

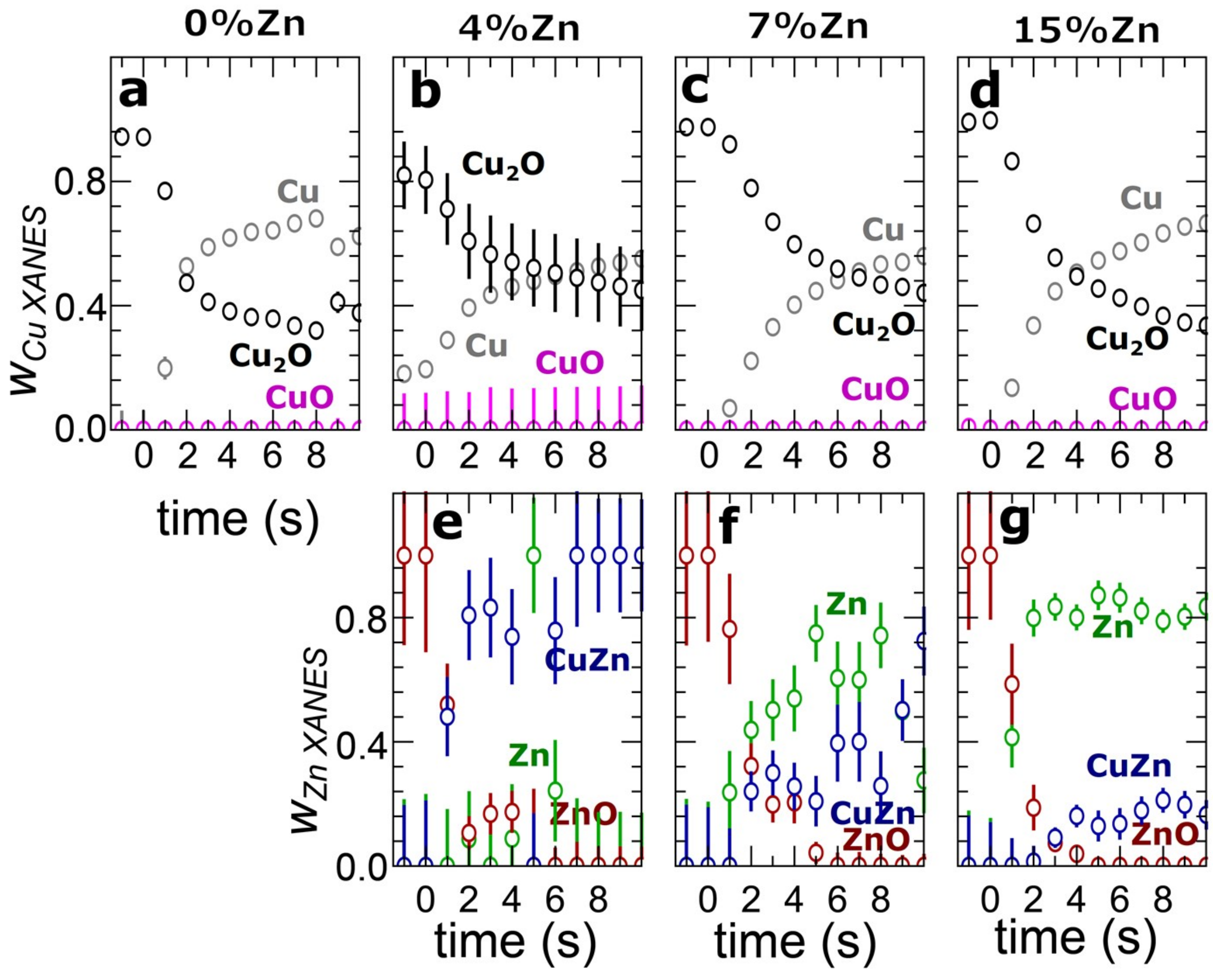

Figure S12. LCA of the time-dependent Cu K-edge (a-c) and Zn K-edge (d-f) XANES data for Zn/Cu_2O NCs samples with Zn loading as indicated in the figure. The analyzed spectra were collected during the first 10 seconds of the CO_2RR at a current density of -500 mA/cm² (e-h). The Cu K-edge XANES spectra of the sample with 4% Zn loading may suffer from self-absorption.

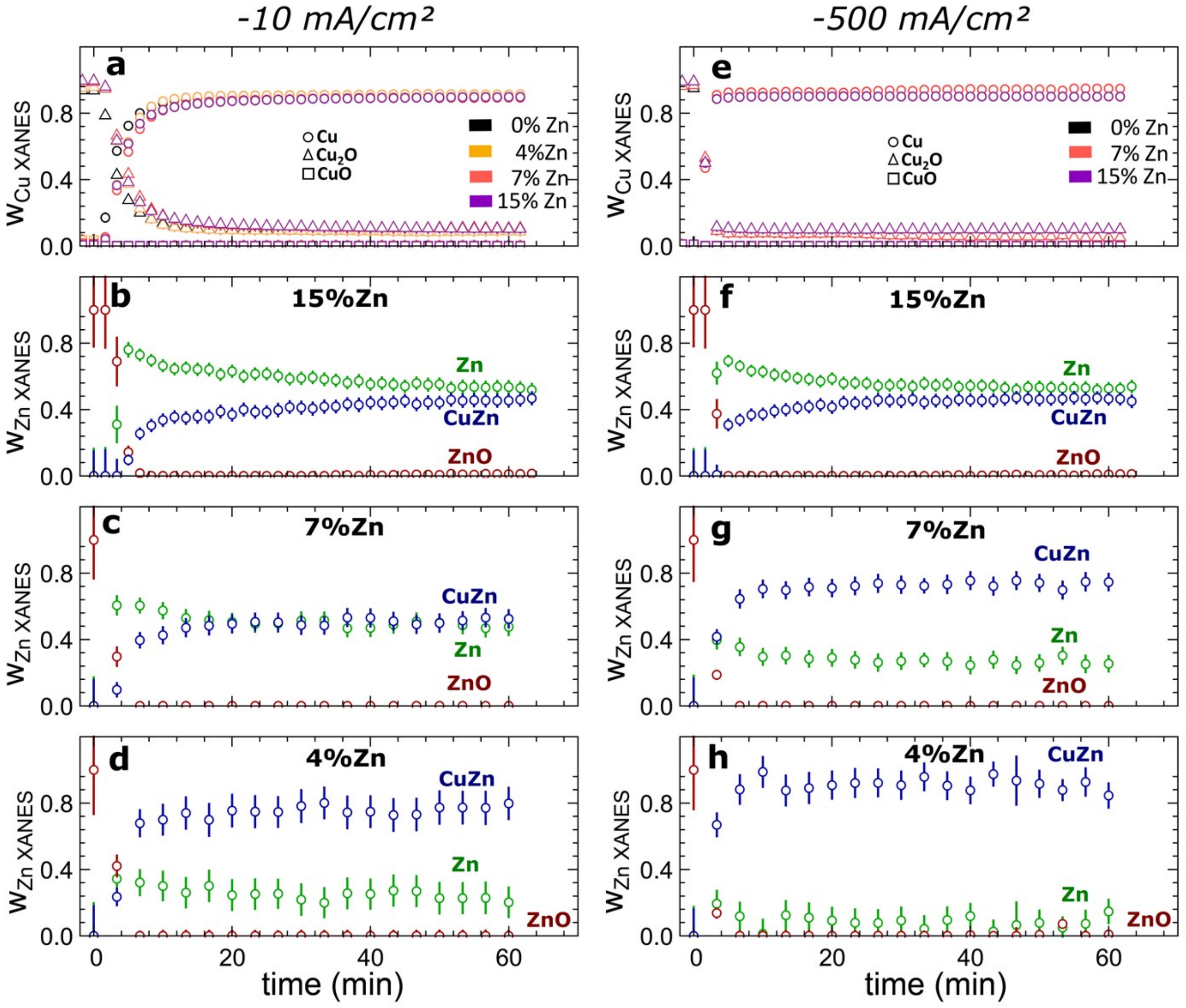

Figure S13. Linear combination analysis (LCA) of the time-dependent Cu K-edge (a,e) and Zn K-edge (b-d, f-h) XANES data for Zn/Cu_2O NCs samples with Zn loadings as indicated in the figure. The analyzed spectra were collected during 1 hour of CO_2RR at a current density of -10 mA/cm² (a-d) and -500 mA/cm² (e-h). The Cu K-edge XANES spectra of the sample with 4% Zn loading that was collected at a current density of -500mA/cm² may suffer from self-absorption, and therefore, was not included in this figure.

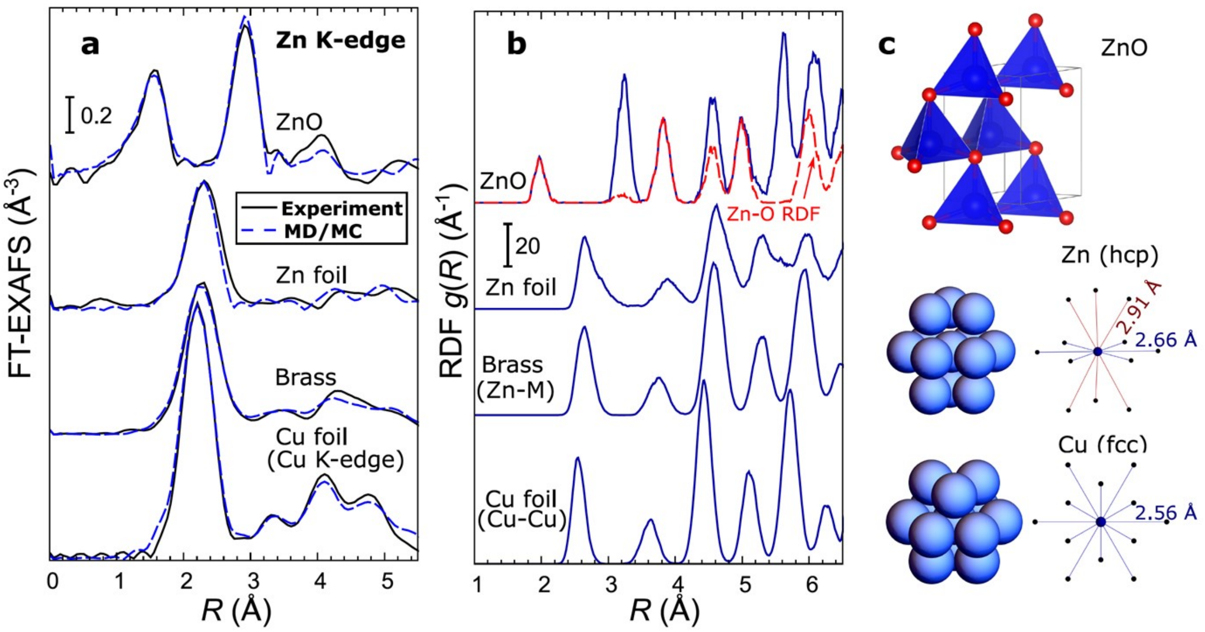

Figure S14. (a) Comparison of experimental Fourier-transformed Zn K-edge EXAFS spectra for wurtzite-type ZnO, Zn foil and brass foil, and Cu K-edge EXAFS spectra for Cu foil with the theoretical spectra, which were obtained in molecular dynamics (for ZnO) and Monte Carlo (for metals) simulations using empirical force field models. The spectra are shifted vertically for clarity. (b) Here, the total radial distribution functions (RDFs) obtained in MD/MC simulations for bulk reference materials are displayed. The partial contribution of Zn-O bonds in ZnO is shown separately. The RDFs are shifted vertically for clarity. (c) Schematic depiction of the wurtzite-type ZnO structure (red spheres – oxygen atoms, blue spheres – Zn atom), the distorted hcp-type structure of metallic Zn and the fcc-type structure of metallic Cu.

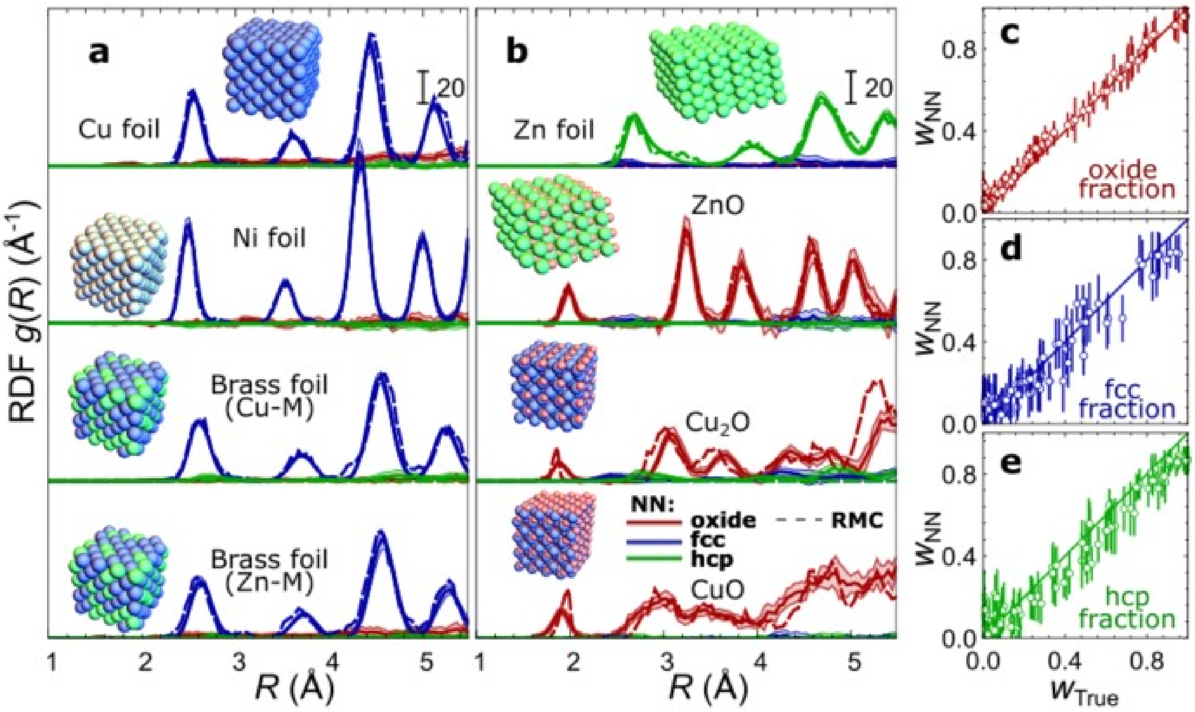

Figure S15. Validation of the neural network (NN) accuracy for reference compounds, using reverse Monte Carlo (RMC) methods. (a,b) The partial RDFs, extracted by the NN for oxide, fcc and hcp phases from experimental Cu K-edge EXAFS spectra for Cu foil, CuZn brass foil, Cu_2O and CuO oxides, and from Zn K-edge EXAFS spectra for Zn foil, CuZn brass foil and ZnO oxide are compared with the RDFs, obtained from the same data using the RMC method. RMC calculations are taken from Ref.[6] Insets show the final structure models obtained from the RMC simulations. The RDFs are shifted vertically for clarity. (c,d,e) Weights of oxide (c), fcc-like (d) and hcp-like (e) phases, obtained by the NN-EXAFS methods for artificial mixtures of experimental Zn K-edge EXAFS spectra for ZnO, CuZn brass foil and Zn foil. The obtained weights are compared with the known true weights of the corresponding reference spectra in the analyzed mixture.

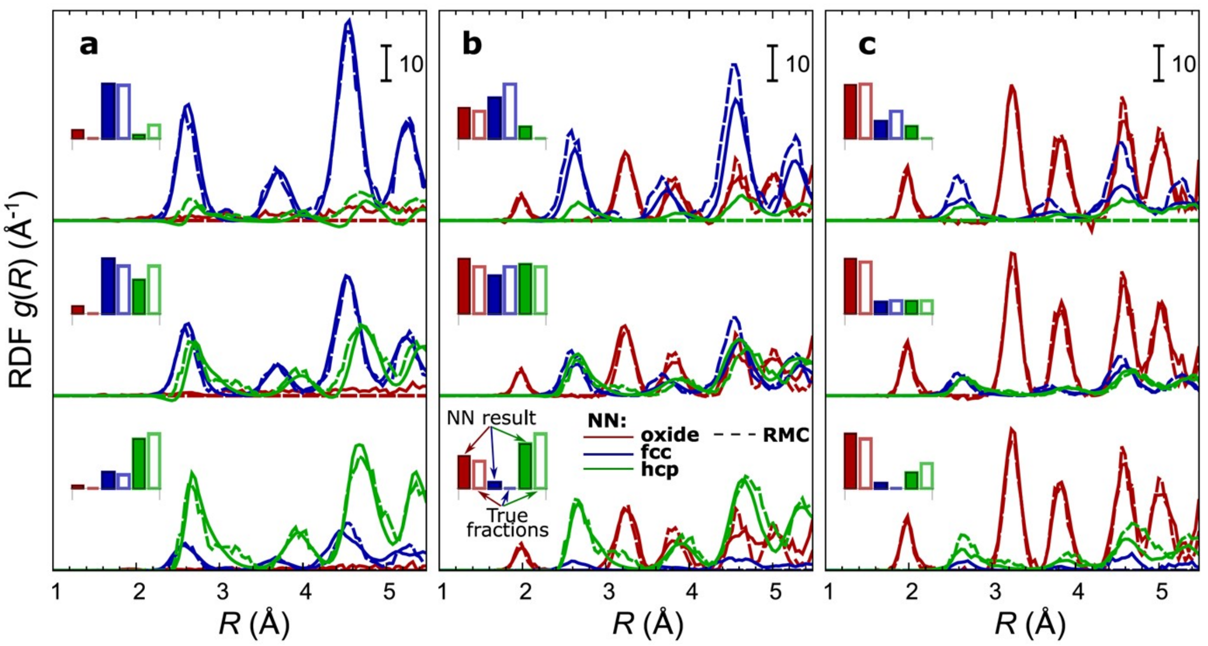

Figure S16. Examples of RDFs of oxide, fcc-like and hcp-like phases, obtained by the NN-EXAFS methods for artificial mixtures of experimental Zn K-edge EXAFS spectra for ZnO, CuZn brass foil and Zn foil. The obtained RDFs (solid lines) are compared with the corresponding mixture of RDFs from RMC simulations (dashed lines) for the same experimental data. The RDFs are shifted vertically for clarity. In the insets, corresponding weights of oxide, fcc-like and hcp-like phases, obtained by the NN-EXAFS method (filled bars), are compared with the known true weights of the corresponding reference spectra in the analyzed mixture (open bars).

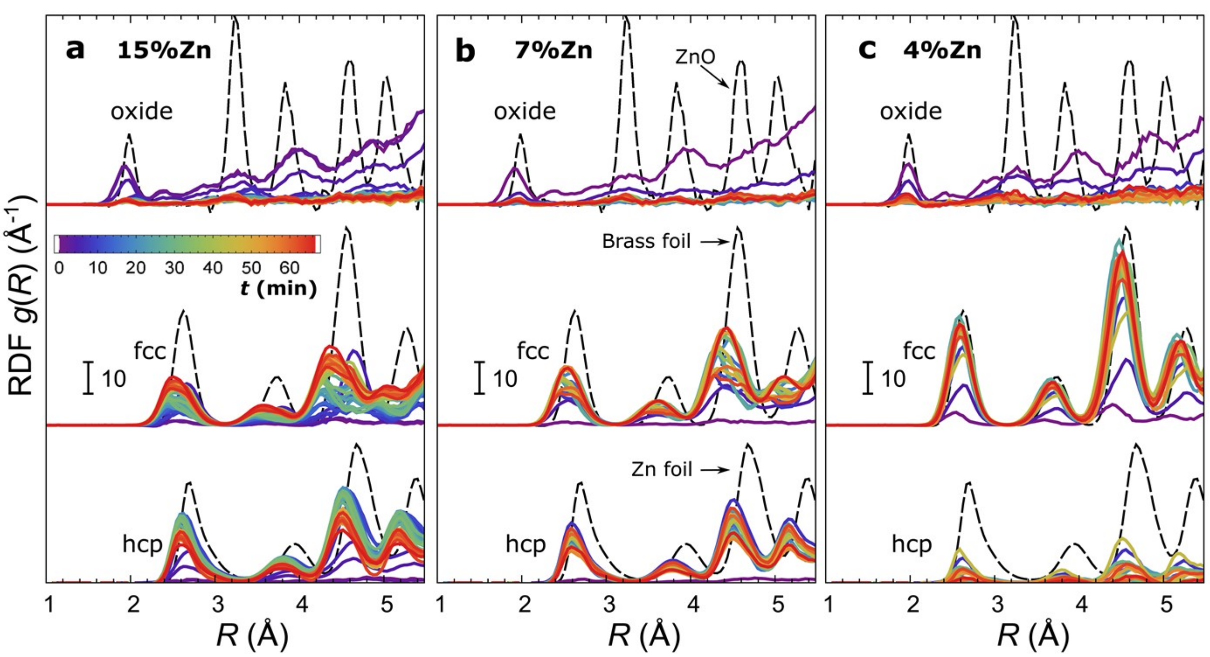

Figure S17. The time-dependent evolution of the RDFs obtained by the NN-EXAFS analysis from *operando* Zn K-edge EXAFS data for bimetallic CuZn catalysts with (a) 15%, (b) 7% and (c) 4% Zn content during CO_2RR at a current density of -10 mA/cm^2. The partial RDFs corresponding to an oxide phase, a metallic phase with fcc-type structure and a metallic phase with non-fcc type structure are shown. The RDFs are shifted vertically for clarity and the RDFs for reference materials (ZnO, brass foil and Zn foil) are shown for comparison.

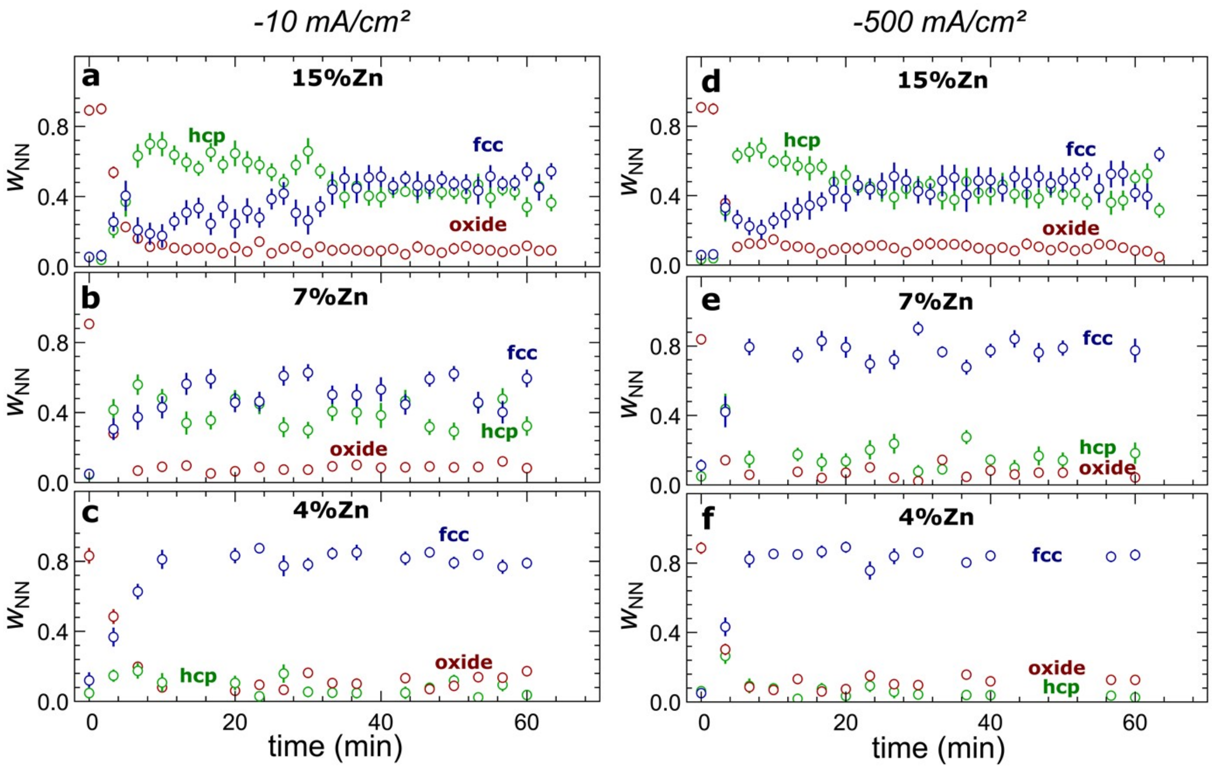

Figure S18. The evolution of the relative concentrations of oxide, fcc-like and hcp-like phases, as obtained by the NN from operando Zn K-edge EXAFS data for bimetallic catalysts with a Zn loading of (a,d) 15%, (b,e) 7% and (c,f) 4% during CO_2RR at -10 mA/cm^2 (a-c) and -500 mA/cm^2 (d-f) current density.

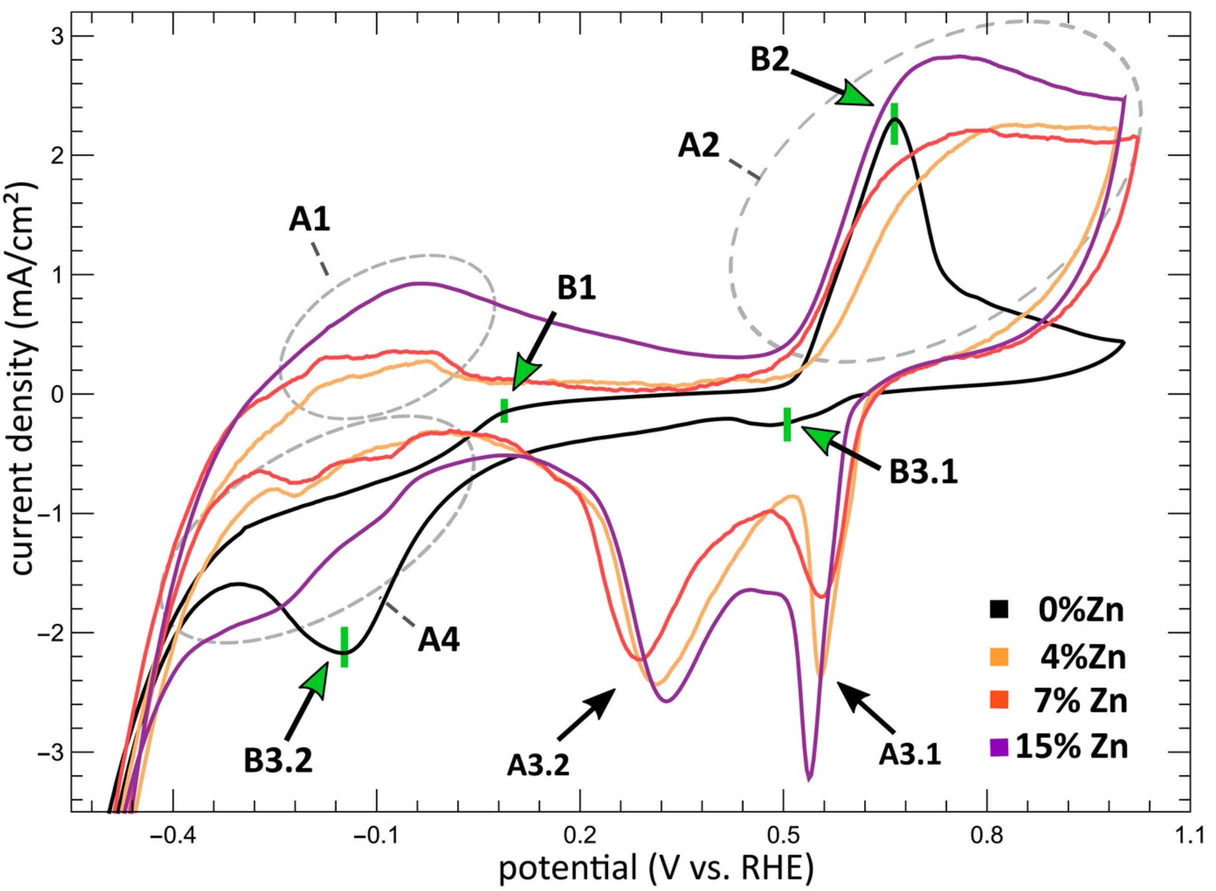

Figure S19. Cyclic voltammetry (CV) scans for the Cu_2O NCs and bimetallic Zn/Cu_2O NC catalysts with different Zn loadings. The current density is plotted as a function of the potential. The CV peaks for the bimetallic CuZn samples are labeled as A1, A2, A3.1, A3.2 and A4, while the CV peaks appearing in the pure Cu_2O catalyst are denoted as B1, B2, B3.1 and B3.2.

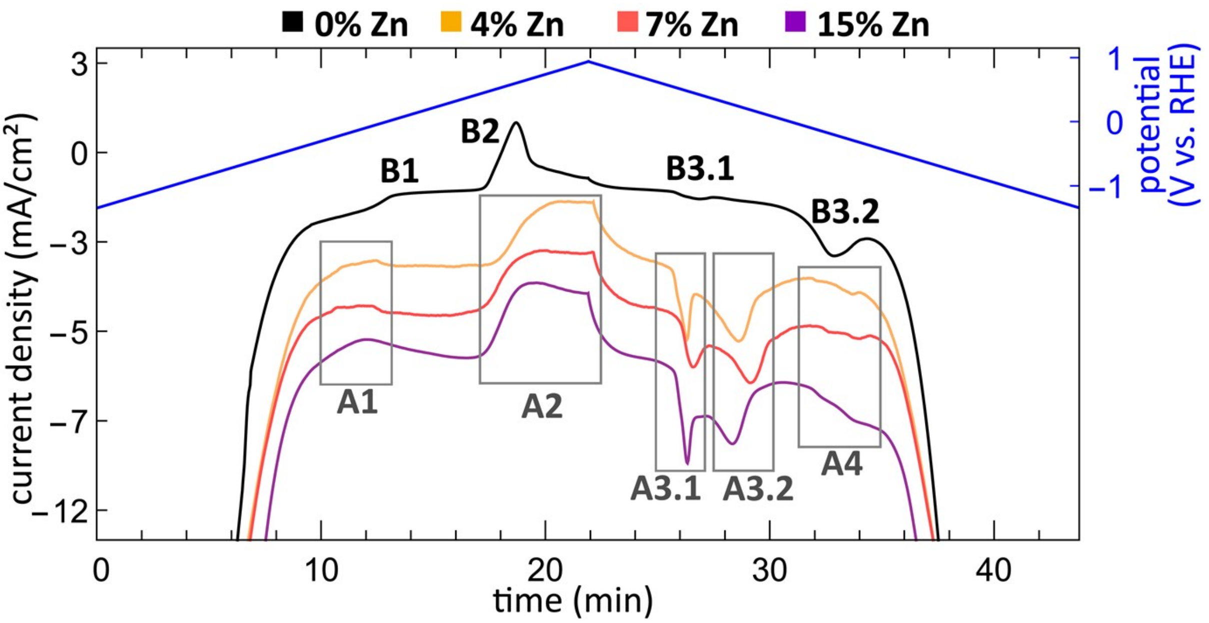

Figure S20. CVs for all samples with the current density plotted as a function over time. The scans are shifted vertically for clarity.

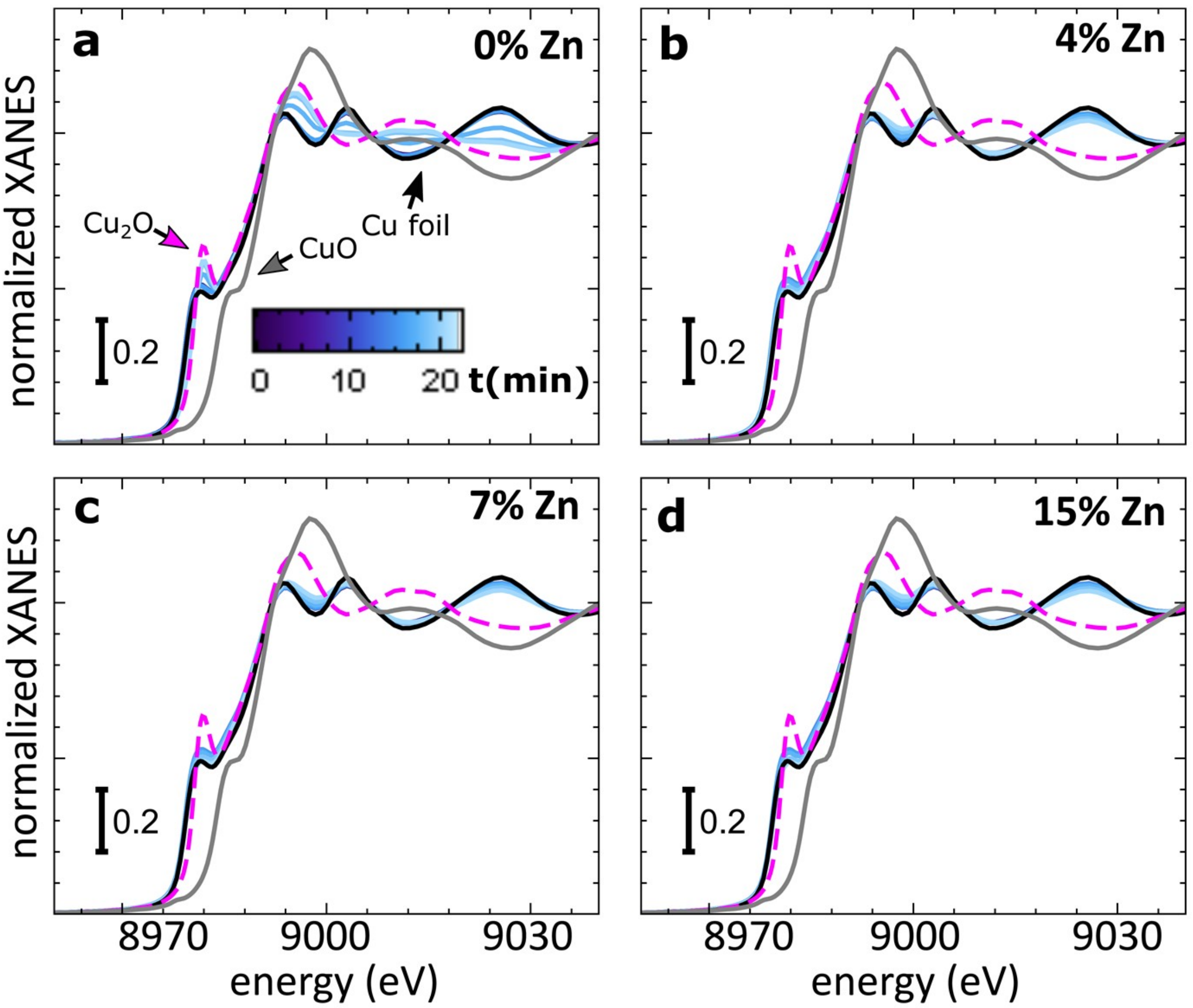

Figure S21. The evolution of the Cu K-edge XANES (a-d) spectra for pure Cu_2O NC catalyst (a) and Zn/Cu_2O NC catalysts with Zn loadings of 4% (b), 7% (c) and 15% (d) during the anodic part of the CV scan. Reference Cu K-edge spectra for bulk Cu_2O and Cu foil are shown for comparison (dashed magenta line and solid black line, respectively).

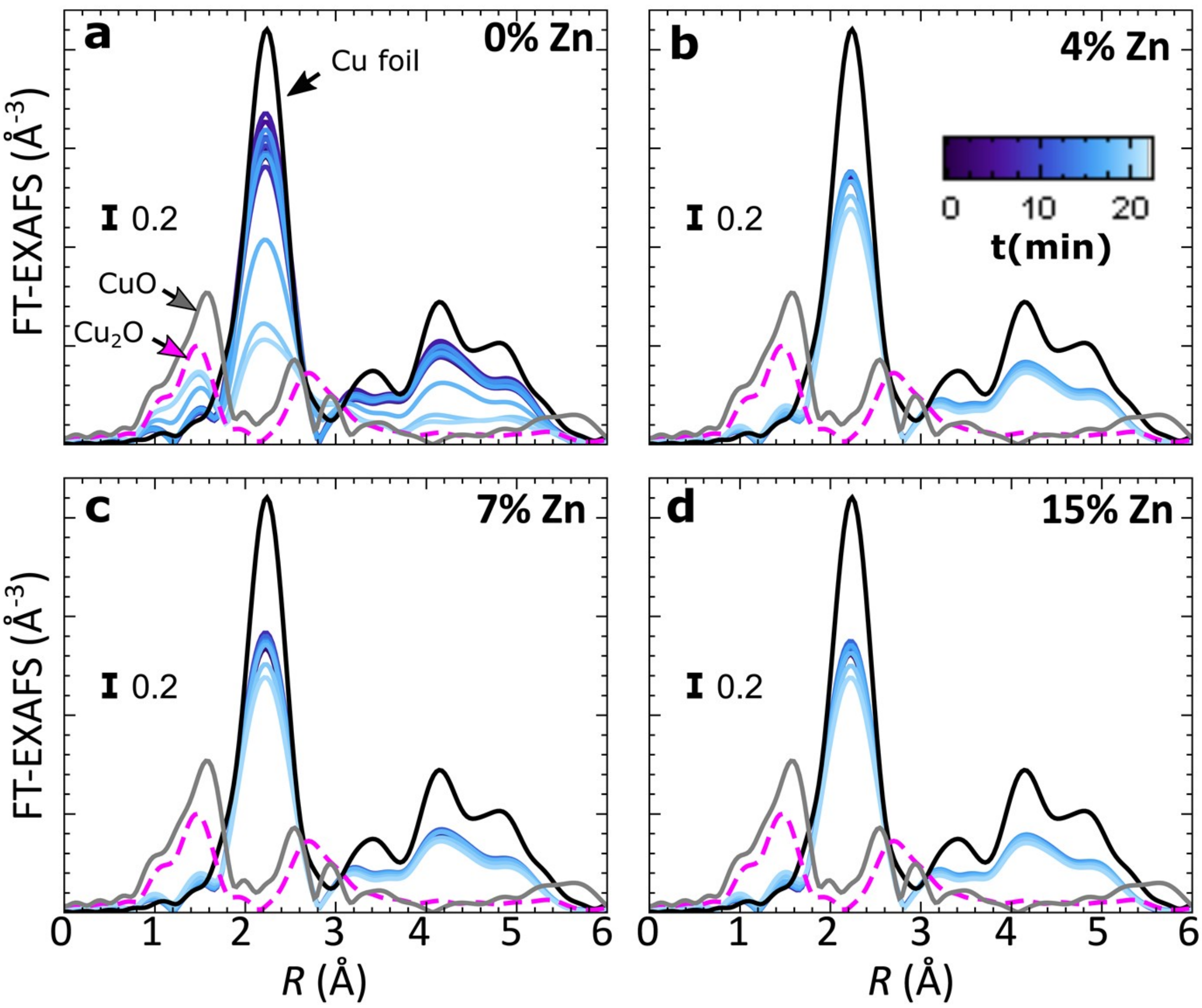

Figure S22. The evolution of the Cu K-edge FT-EXAFS (a-d) spectra for pure Cu_2O NC catalyst (a) and Zn/Cu_2O NC catalysts with Zn loadings of 4% (b), 7% (c) and 15% (d) during the anodic part of the CV scan. Reference Cu K-edge spectra for bulk Cu_2O and Cu foil are shown for comparison (dashed magenta line and solid black line, respectively).

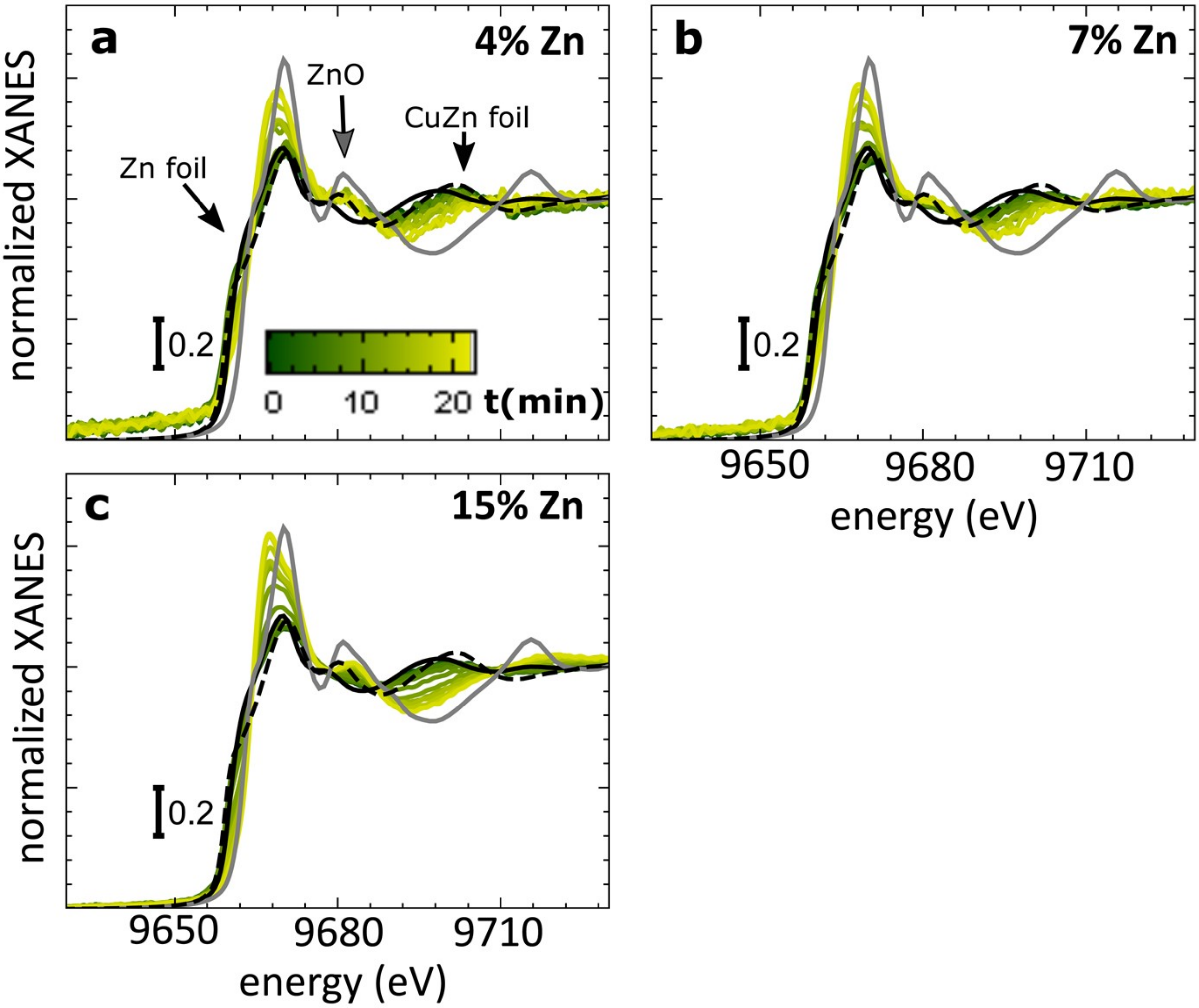

Figure S23. The evolution of the Zn K-edge XANES (a-c) spectra for Zn/Cu_2O NC catalysts with Zn loadings of 4% (a), 7% (b) and 15% (c) during the anodic part of the CV scan. The reference Zn K-edge spectra for bulk ZnO, CuZn brass foil and Zn foil are shown for comparison (solid gray line, dashed black line and solid black line, respectively).

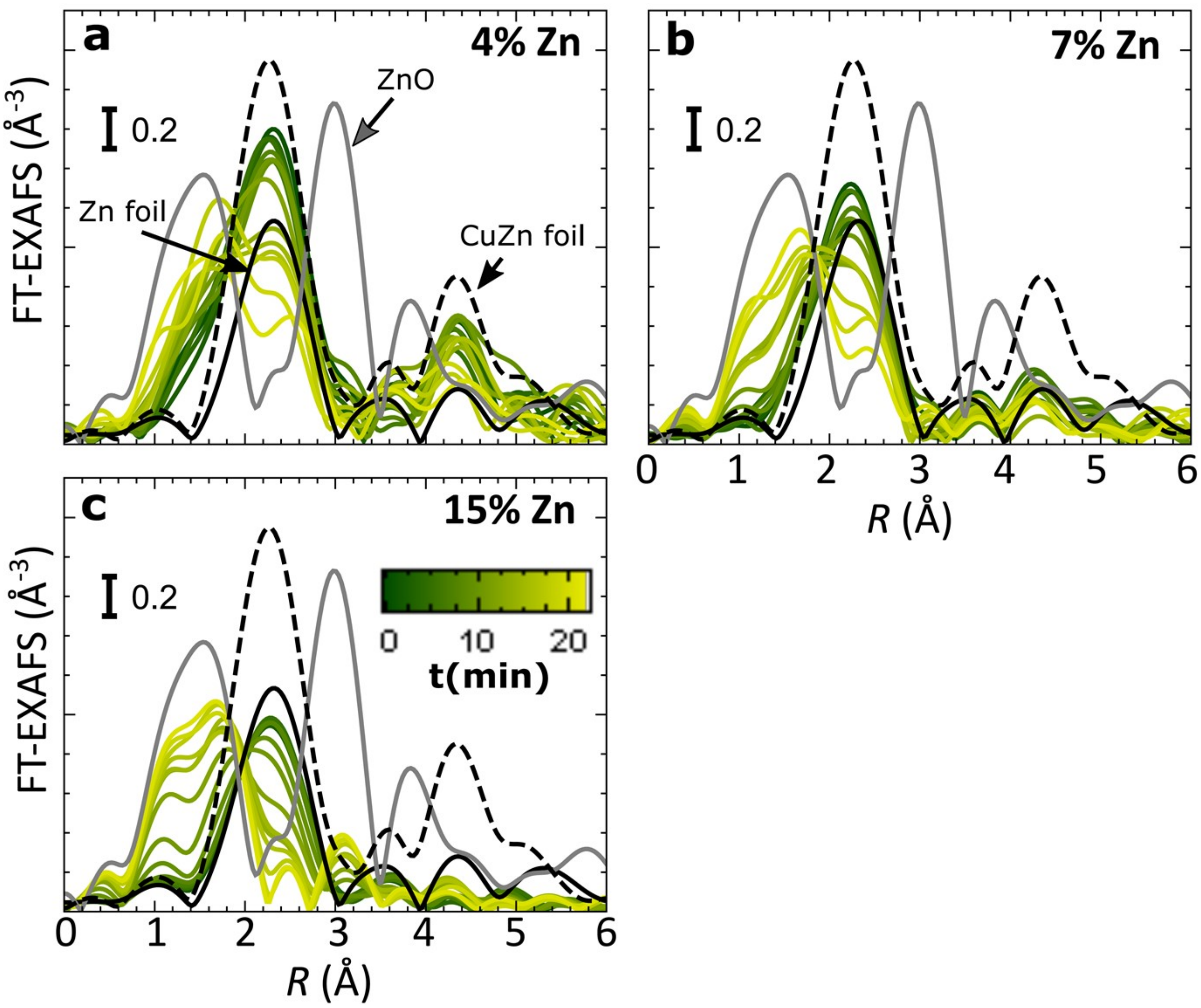

Figure S24. The evolution of the Zn K-edge FT-EXAFS (a-c) for Zn/Cu_2O NC catalysts with Zn loadings of 4% (a), 7% (b) and 15% (c) during the anodic part of the CV scan. The reference Zn K-edge spectra for bulk ZnO, CuZn brass foil and Zn foil are shown for comparison (solid gray line, dashed black line and solid black line, respectively).

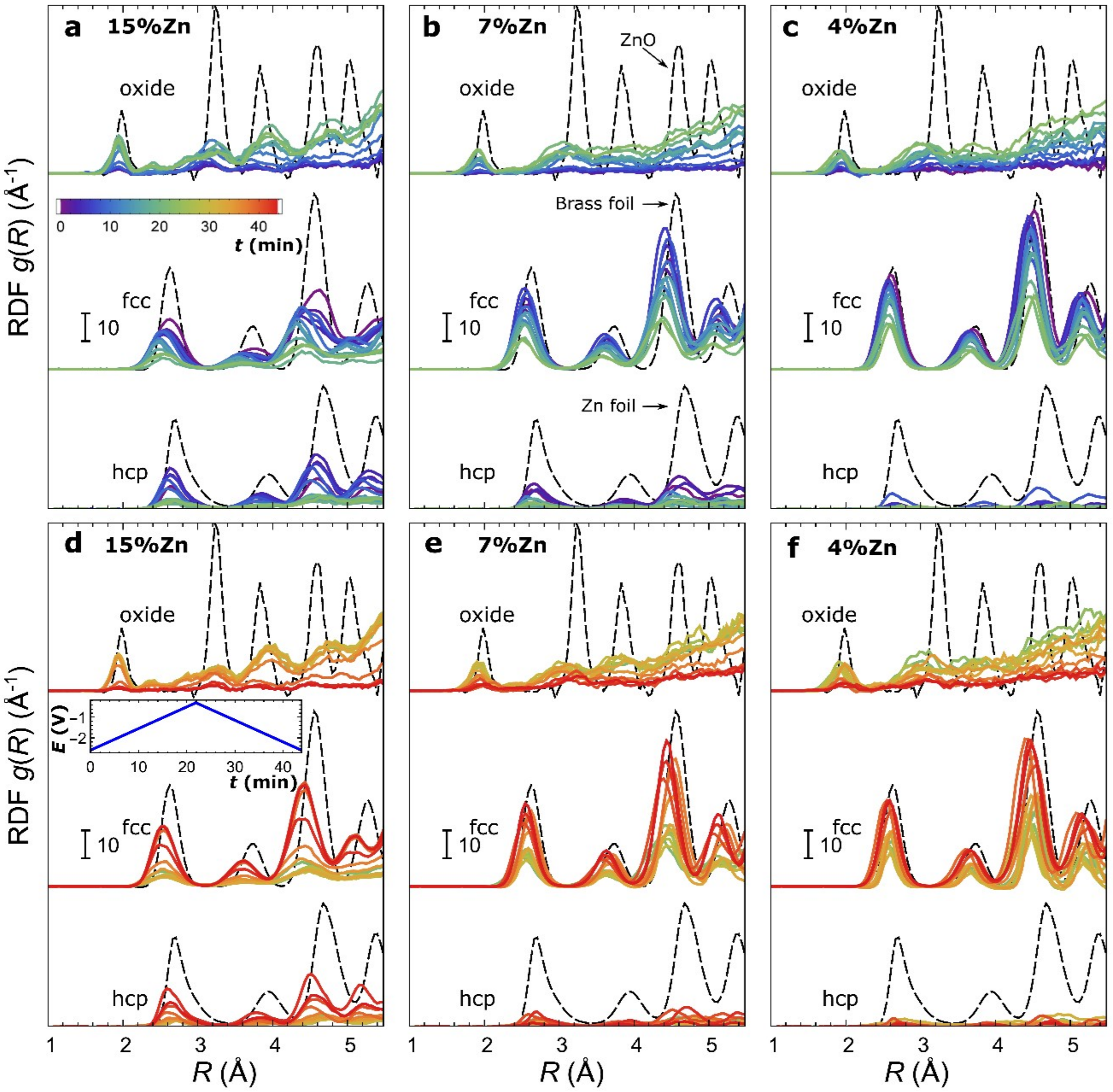

Figure S25. The evolution of the RDFs for oxide, fcc-like and hcp-like phases, as obtained by the NN from *operando* Zn K-edge EXAFS data for bimetallic catalysts with (a,d) 15%, (b,e) 7% and (c,f) 4% Zn loading during the CV scan. Panels (a-c) show the evolution of the RDFs during the anodic part of the CV (increasing potential), while panels (d-f) show the evolution of RDFs during the cathodic part of the CV (decreasing potential). The RDFs are shifted vertically for clarity. The RDFs for reference materials (ZnO, brass foil and Zn foil) are shown for comparison. The inset in (d) shows the time-dependency of the applied potential.

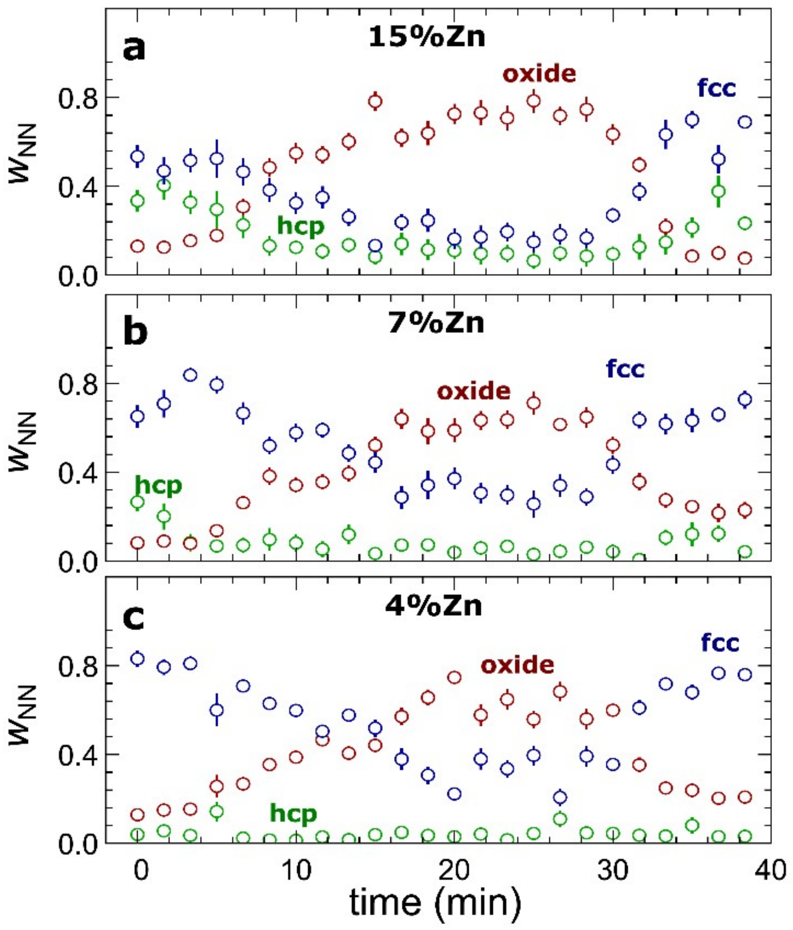

Figure S26. The evolution of the relative concentrations of oxide, fcc-like and hcp-like phases, as obtained by the NN from *operando* Zn K-edge EXAFS data for bimetallic catalysts with (a) 15%, (b) 7% and (c) 4% Zn loading during the CV scan.

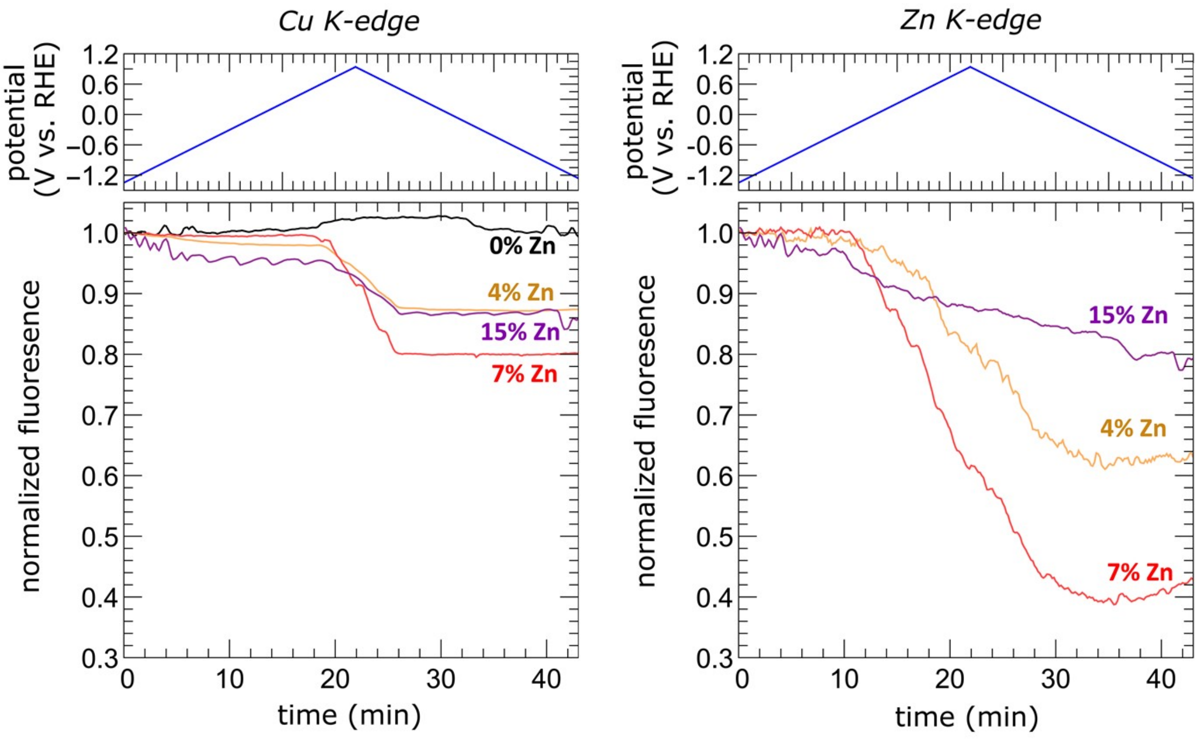

Figure S27. Time-dependent evolution of the jump in the X-ray fluorescence signal at (a) the Cu K-edge and (b) the Zn K-edge for all samples during the CV. A decrease of the fluorescence signal indicates the dissolution of unstable Cu(II) and Zn(II) species. Data are normalized, so that the fluorescence jump is equal to 1 at the beginning of the CV scan.

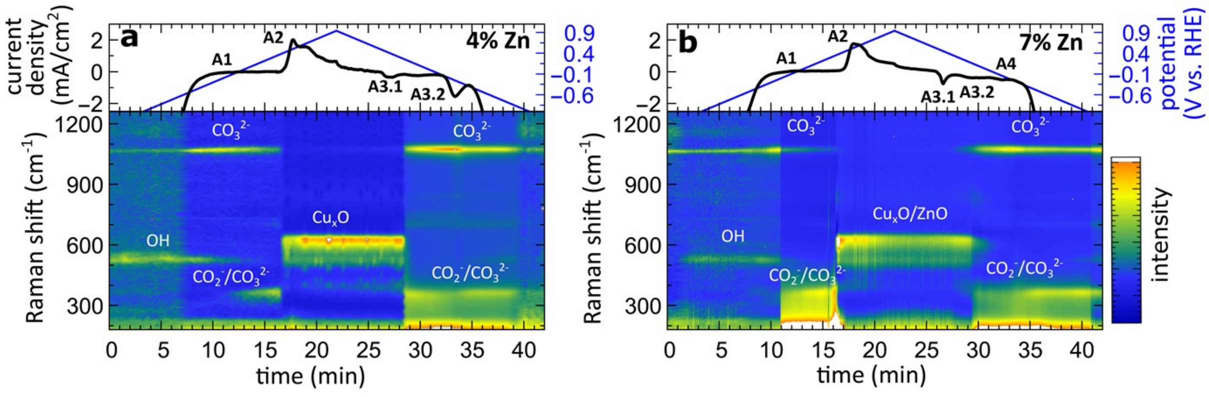

Figure S28. Time-dependent SERS band shifts during the CV scan, and the corresponding changes in the current density for (a) Zn/Cu_2O NCs catalyst with a 4% Zn loading (b) Zn/Cu_2O NCs catalyst with a 7% Zn loading. In panel a, the sample was accidentally exposed to higher potentials, where the Zn(II) species are unstable. Therefore, we suspect that the amount of Zn present during the reduction cycle might be less than 4% in this sample. This, however, should not affect the main CV features, corresponding to the oxidation of copper as well as the reduction of surface Cu species.

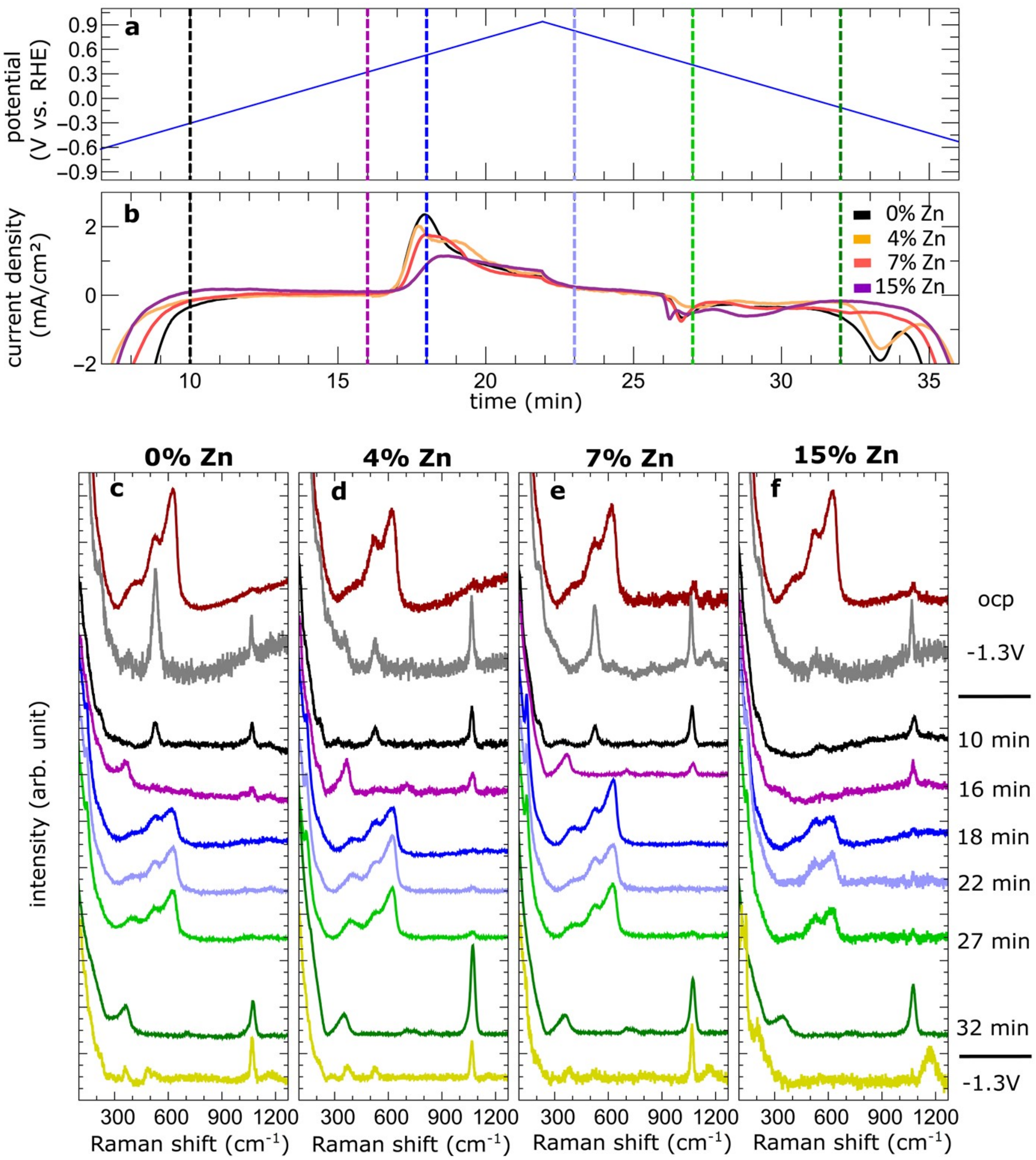

Figure S29. Selected Raman spectra collected at different potentials (a), namely at open circuit potential, during the CV scan (b) and before and after the CV at a potential relevant for the CO_2RR (-1.3V vs. RHE), (c) for the monometallic sample and (d-f) for the bimetallic samples. The spectra are shifted vertically for clarity.

Supplementary References

1. X.-W. Liu, F.-Y. Wang, F. Zhen and J.-R. Huang, *RSC Advances*, 2012, **2**, 7647-7651.
2. A. Herzog, A. Bergmann, H. S. Jeon, J. Timoshenko, S. Kühl, C. Rettenmaier, M. Lopez Luna, F. T. Haase and B. Roldan Cuenya, *Angewandte Chemie International Edition*, 2021, **60**, 7426-7435.
3. H. S. Jeon, J. Timoshenko, C. Rettenmaier, A. Herzog, A. Yoon, S. W. Chee, S. Oener, U. Hejral, F. T. Haase and B. Roldan Cuenya, *Journal of the American Chemical Society*, 2021, **143**, 7578-7587.
4. B. Bornmann, J. Kläs, O. Müller, D. Lützenkirchen-Hecht and R. Frahm, *AIP Conference Proceedings*, 2019, **2054**, 040008.
5. B. Ravel and M. Newville, *Journal of Synchrotron Radiation*, 2005, **12**, 537-541.
6. J. Timoshenko, H. S. Jeon, I. Sinev, F. T. Haase, A. Herzog and B. Roldan Cuenya, *Chemical Science*, 2020, **11**, 3727-3736.
7. U. Hejral, J. Timoshenko, D. Kordus, M. Lopez Luna, N. J. Divins, S. Widrinna, I. Zegkinoglou, L. Pielsticker, H. Mistry, J. Anibal Boscoboinik, S. Kuehl and B. Roldan Cuenya, *Journal of Catalysis*, 2021.
8. J. Timoshenko and A. Kuzmin, *Computer Physics Communications*, 2009, **180**, 920-925.
9. S. J. Gurman and R. L. McGreevy, *Journal of Physics: Condensed Matter*, 1990, **2**, 9463-9473.
10. J. Timoshenko, A. Kuzmin and J. Purans, *J Phys Condens Matter*, 2014, **26**, 055401.
11. J. Timoshenko and A. I. Frenkel, *Catalysis Today*, 2017, **280**, 274-282.
12. J.-J. Velasco-Vélez, K. Skorupska, E. Frei, Y.-C. Huang, C.-L. Dong, B.-J. Su, C.-J. Hsu, H.-Y. Chou, J.-M. Chen, P. Strasser, R. Schlögl, A. Knop-Gericke and C.-H. Chuang, *The Journal of Physical Chemistry B*, 2018, **122**, 780-787.
